# Clinicopathological and Prognostic Significance of Inhibitor of Apoptosis Protein (IAP) Family Members in Lung Cancer: A Meta-Analysis

**DOI:** 10.3390/cancers13164098

**Published:** 2021-08-14

**Authors:** Stephen Fung, Wolfram Trudo Knoefel, Andreas Krieg

**Affiliations:** Department of Surgery (A), Heinrich-Heine-University and University Hospital Duesseldorf, 40225 Duesseldorf, Germany; Stephen.Fung@med.uni-duesseldorf.de (S.F.); knoefel@med.uni-duesseldorf.de (W.T.K.)

**Keywords:** lung cancer, SCLC, NSCLC, inhibitors of apoptosis, BIR domain, survivin, XIAP, livin, BRUCE

## Abstract

**Simple Summary:**

Lung cancer is the leading cause of cancer-related death worldwide. Although novel therapy regimens using immuno- and targeted therapy have improved survival for a subgroup of patients with lung cancer, the five-year survival rate is still poor. The inhibitor of apoptosis protein (IAP) family represents a heterogeneous group of anti-apoptotic proteins that are highly expressed in a variety of human malignancies. Despite conflicting results regarding the prognostic significance of IAPs, high expression of some members of this family have been extensively reported to be associated with poor prognosis in lung cancer patients. Therefore, there might be a subgroup of patients that could benefit from a targeted therapy against specific IAP family members in lung cancer. The aim of this study was to perform a meta-analysis to investigate the prognostic value of IAP family members and their association with clinicopathological features in lung cancer.

**Abstract:**

Lung cancer is the most common cause of cancer-related death worldwide. Approximately 85% is non-small-cell and 15% is small-cell lung cancer. The inhibitor of apoptosis proteins (IAPs) represent a heterogeneous family of anti-apoptotic proteins, some members of which have been reported to correlate with clinical outcome in lung cancer. We screened PubMed, Web of Science, and Scopus for studies that investigated the prognostic value and clinicopathological features of IAPs in lung cancer. Forty-five eligible studies with 4428 patients assessed the expression of the IAPs survivin, XIAP, livin, and BRUCE. The pooled hazard ratio (HR) of 33 studies that analyzed overall survival (OS) revealed a positive correlation between survivin expression and poor prognosis. Seven studies displayed a strong association between survivin and disease recurrence. Two studies that assessed the expression of XIAP and livin, respectively, proved a significant relationship of these IAPs with poor OS. Meta-analyses of clinicopathological variables revealed a significant association between survivin and T stage, UICC stage, the presence of lymph node metastasis, and grade of differentiation. In conclusion, high expression of distinct IAPs significantly correlates with prognosis in lung cancer. Therefore, lung cancer patients might benefit from a targeted therapy against specific IAPs.

## 1. Introduction

Lung cancer is the most common cause of cancer-related death worldwide [1]. According to the World Health Organization (WHO) and International Association for the Study of Lung Cancer (IASLC), lung cancer can be classified as small-cell lung cancer (SCLC) and non-small-cell lung cancer (NSCLC) [2,3,4]. Among all cases, approximately 85% comprises NSCLC and 15% SCLC [2,5,6,7]. To date, surgical resection according to oncological principles remains the mainstay in the treatment of early-stage lung cancer (stage I or II) [2,8,9]. However, more than 60% of patients with lung cancer present with locally advanced or metastatic disease (stage III or IV) at the time of diagnosis, limiting surgical resection as a primary treatment option [9]. In addition, even after complete surgical resection of early-stage NSCLC, a high risk of disease recurrence has been reported [5]. Although multimodal platinum-based chemotherapy, radiation, and surgery, when possible, are well established in the treatment of patients with advanced NSCLC, locally advanced NSCLC is still associated with poor survival [10]. Over the past decade, targeted therapy with tyrosine kinase inhibitors (TKIs) such as erlotinib or gefitinib and anaplastic lymphoma kinase inhibitors (ALK) such as crizotinib have improved clinical outcome in a subset of lung cancer patients whose tumors harbor EGFR (epidermal growth factor receptor) and EML4-ALK (echinoderm microtubule-associated protein-like 4 and anaplastic lymphoma kinase) alterations, respectively [11,12,13]. Specifically for NSCLC with reported EGFR mutation rates between 7% to 37% in Caucasian and 40% to 64% in Asian patients, EGFR-sensitizing mutations were identified as oncogenic drivers predicting response to EGFR TKIs [13]. In NSCLC cases with EGFR mutations, approximately 90% harbor an exon 19 deletion or exon 21 L858R mutation, which renders the tumors sensitive to EGFR TKIs [13,14,15,16,17]. Thus, due to their increased benefit on progression-free survival (PFS) and mild adverse effects, certain FDA- (US Food and Drug Administration) and EMA- (European Medicines Agency) approved EGFR TKIs such as erlotinib and gefitinib are usually implemented as first-line therapy for patients with advanced NSCLC [14,17,18,19]. However, emerging resistance to EGFR TKI therapy substantially affects five-year survival rates, especially for patients with metastatic lung disease [20]. Therefore, novel therapeutic strategies are needed to improve patient outcomes. Recently, a dual EGFR-VEGF pathway inhibition (e.g., erlotinib and bevacizumab or erlotinib and ramucirumab) has been reported as a promising strategy for patients with EGFR-mutant NSCLC [13].

More recently, immunotherapy using PD-L1 (programmed cell death ligand 1) as a key immunoregulatory molecule has been developed and is increasingly used in the treatment of lung cancer patients [21,22]. As a key immunoregulatory molecule, PD-L1 interacts with its immune checkpoint receptor PD-1 (programmed cell death protein 1), which is expressed on the surface of macrophages and activated T cells and B cells. The binding of PD-L1 to PD-1 enhances immunosuppression and avoids tumor-induced immune destruction by inhibiting the proliferation and survival of cytotoxic T cells and reducing cytokine production [23,24,25]. In NSCLC patients, positive PD-L1 expression is observed in 50% to 70% of cases [23]. Recently, expression of PD-L1 was reported to be associated with increased tumor proliferation and aggressiveness, as well as shorter survival times for patients with NSCLC [26]. The evolvement of immune checkpoint inhibitors (ICI) based on PD-1 inhibition (e.g., nivolumab, pembrolizumab) and PD-L1 inhibition (durvalumab, atezolizumab, and avelumab) have significantly improved the survival rates and clinical outcomes in patients with metastatic NSCLC [23,27]. Moreover, in first-line and second-line settings, ICI monotherapy and dual therapy or a therapeutic approach in combination with chemotherapy has displayed an overall survival benefit compared to standard platinum-based regimens [23,27,28,29]. However, not all patients experience favorable response to treatment with ICI due to severe side effects and underlying clinical condition [30]. Although the development of targeted therapy has improved the clinical outcome in a subgroup of lung cancer patients, the five-year survival rate is still less than 20% [2,9,31]. Therefore, there is a need to detect novel molecular markers that might positively impact the outcome of patients with lung cancer. One of the most important targets of cancer therapies is the induction of apoptotic cell death. The molecular mechanisms by which cancer drugs induce apoptosis can be mediated via the receptor depending extrinsic or mitochondrial intrinsic pathway, resulting in the activation of caspases, a family of cysteine-rich proteases [32]. Unfortunately, evasion of apoptotic cell death is a well-known phenomenon in tumor cells and leads to chemotherapeutic resistance. A common molecular mechanism by which tumor cells achieve resistance to chemotherapeutic agents is the overexpression of anti-apoptotic proteins. Among these anti-apoptotic proteins, the inhibitor of apoptosis protein (IAP) family members have been extensively investigated during the last decades. The IAP family represents a heterogeneous group of proteins that are highly expressed in a variety of human malignancies [33,34,35,36,37]. This family consists of eight members, namely, neuronal IAP (NAIP/BIRC1), cellular IAPs (cIAP1/BIRC2, cIAP2/BIRC3), X-linked IAP (XIAP/BIRC4), survivin/BIRC5, BIR repeat containing ubiquitin-conjugating enzyme (BRUCE/BIRC6), livin/BIRC7, and testis specific IAP (Ts-IAP/BIRC8) [38]. Each member contains at least a Baculovirus IAP Repeat (BIR) domain of approximately 70 amino acids that is responsible for the inhibitory properties [39]. Although the number of BIR domains varies among the IAP family members, each BIR domain consists of cysteine and histidine residues in a well-defined pattern, which represents a novel zinc-binding fold [40].

**Figure 1:**
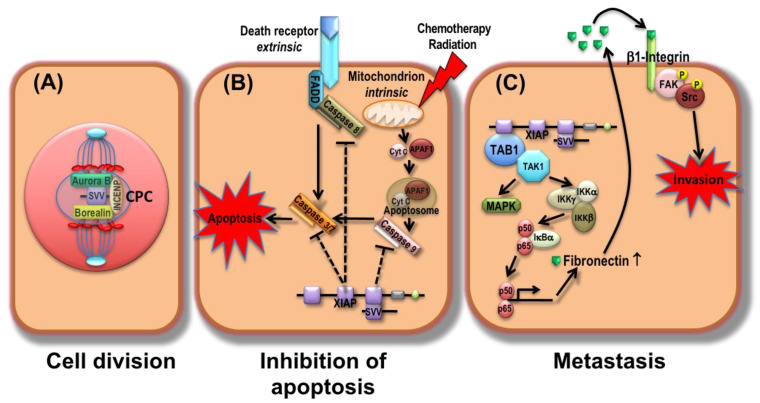
Schematic representation of survivin actions. (**A**) During cell division, survivin (SVV) forms with Aurora B, Borealin, and INCENP (inner centromere protein), the chromosomal passenger complex (CPC) that regulates chromosome segregation and cytokinesis. By directly binding, survivin protects XIAP from proteosomal degradation. (**B**) The stabilized XIAP-survivin complex inhibits apoptosis mediated by caspases after the activation of intrinsic or extrinsic signaling pathways. FADD, FAS-associated death domain protein; Cyt c, cytochrome c; APAF1, apoptotic protease activating factor 1. (**C**) XIAP and survivin cooperate to activate transcription factor NF-κB via the TAB1/TAK1 complex, which induces the overexpression of fibronectin 1. This leads to an autocrine or paracrine stimulation of β1-integrin and phyosphorylation (P) of proto-oncogene tyrosine kinase Src and focal adhesion kinase FAK. Activation of these cell motility kinases induces metastasis by activating tumor cell invasion.

Compared to other IAPs, survivin, encoded by the BIRC5 gene, is the smallest and most extensive studied member of the IAP family in lung cancer. Although it contains a single N-terminal BIR domain and a C-terminal coiled coil domain, survivin consists of four exons and three introns covering 14,796 nucleotides on chromosome 17q25, forming transcripts with different functional domains [40]. It is highly expressed in most human tumors but almost undetectable in normal, fully differentiated adult tissues. It is predominantly present in the cytosol of malignant cells, but nuclear expression has also been demonstrated [41,42,43]. During cell proliferation and division, survivin forms an integral component of the chromosomal passenger complex (CPC), which ensures proper segregation of chromosomes and cytokinesis and thus acts as key mediator of cell proliferation (Figure 1A) [44]. Whereas nuclear survivin is involved in mitosis, cytoplasmic survivin seems to be responsible for anti-apoptotic activity. In this context, survivin binds directly to XIAP, thus preventing XIAP from proteosomal degradation. Consequently, the stabilized XIAP-survivin complex inhibits apoptotic cell death by inhibiting caspase activity (Figure 1B) [45]. Besides its anti-apoptotic function, the XIAP-survivin complex induces tumor cell invasion and metastasis by altering focal adhesion via an interaction with the TAB1/TAK1 complex, which activates Nuclear Factor kappa B (NF-κB) (Figure 1C) [46]. Moreover, transcription of survivin has been demonstrated to be regulated by p53, retinoblastoma (Rb), and transcription factor E2F2 [47]. In this context, it has been previously reported that p53-induced downregulation of survivin was mediated through proteosomal degradation of MDM2, a critical negative regulator of p53 [48]. Interestingly, by protecting endothelial cells of the tumor vasculature against drug-induced damage, survivin may be involved in mediating chemotherapeutic resistance [49]. Due to its distinct roles in the inhibition of apoptosis, regulation of cell cycles, and drug resistance, survivin seems to attract more interest as molecular target in lung cancer compared to the other IAPs.

Nonetheless, high expression of some members of the IAP family has been extensively reported to induce chemoresistance, correlate with clinicopathological parameters, and be associated with poor prognosis in lung cancer patients [37,42,50,51,52,53,54,55]. However, conflicting results have been reported regarding the prognostic significance of IAPs in malignant disease. Therefore, we performed a systematic review and meta-analysis using the Population, Intervention, Comparison, Outcome (PICO) model to clarify the question of whether in patients with lung cancer (P) high expression levels of IAPs (I) are associated with clinicopathological parameters and poor survival (CO).

## 2. Materials and Methods

### 2.1. Literature Search

The PICO model was used to define the research question of our systematic review [56]. In addition, the systematic review and meta-analysis were conducted according to the AMSTAR [57] and PRISMA [58] checklists. We then conducted a literature search via PubMed, Web of Science, and Scopus on July 24, 2021, to find articles that investigated the role of IAPs in lung cancer using Boolean operators to connect our search terms together: lung OR pulmonal OR bronchial OR NSCLC OR SCLC AND tumor* OR cancer OR carcinoma OR neoplasm OR malignancy OR adenocarcinoma OR squamous AND survivin OR XIAP OR X-linked OR IAP OR cIAP OR c-IAP OR livin OR NAIP OR ML-IAP OR ILP OR Apollon OR Bruce OR BIRC* OR baculoviral OR “inhibitor of apoptosis”.

### 2.2. Selection Criteria

All eligible studies that assessed the relationship between the expression of IAP family members and clinicopathological parameters and/or performed survival analysis were extracted. Primarily, abstracts and titles of publications obtained from our initial database search were analyzed by S.F. and A.K. to find exactly those articles that investigated the relationship between IAP family members and clinicopathological parameters and/or survival in lung cancer. After rigorous reading of the abstracts that met the criteria, the full texts were analyzed and included in or excluded from the meta-analysis according to the following criteria: (1) expression of the IAP family member was evaluated in lung cancer either by immunohistochemistry (IHC), fluorescence in situ hybridization (FISH), or reverse transcription and polymerase chain reaction (RT-PCR) analysis; (2) expression levels of the IAP family member was compared with clinicopathological characteristics and/or survival outcomes; (3) hazard ratios (HR) with confidence intervals (CI) of the survival analysis were provided or could be calculated from the presented survival curves; (4) articles were written only in the English language; (5) the papers supplied relevant data that compared the expression of an IAP family member with clinicopathological characteristics and enabled us to calculate the odds ratio (OR); (6) if the same patient group was published in several journals by an author, the study with the most complete data set was selected for our meta-analysis; and (7) studies in which clinical samples others than tissue specimen (e.g., serum, plasma, urine) were analyzed for expression levels of IAPs and studies that presented data from The Cancer Genome Atlas (TCGA) portal were excluded.

### 2.3. Data Extraction

Two independent investigators (S.F. and A.K.) reviewed each article for data extraction. The extracted data were recorded independently by both investigators in separate databases by including the first author’s name, year of publication, country of origin, included tumor stages, histology, number of patients, sex, age, administered therapy, source of histological samples, follow-up data, analyzed biomarker (IAP family member), immunohistochemically stained subcellular localization of the biomarker, laboratory methodology, tumor characteristics, hazard ratio (HR) with confidence interval (CI), and cut-off value. Both investigators compared and discussed the entire dataset and reached an agreement if necessary.

### 2.4. Quality Assessment

To assess the methodological quality, each investigator (S.F. and A.K) independently read and scored each publication according to the scale for biological prognostic factors established by the European Lung Cancer Working Party (ELCWP) [59]. The ELCWP scale consists of 4 domains, namely, scientific design, laboratory methodology, generalizability, and results analysis. In each domain a maximum of 10 points can be scored. By scoring all 4 domains a maximum of 40 points can be achieved. Both investigators (S.F. and A.K.) independently calculated their scores and afterwards compared their results to reach a consensus if necessary. The calculated final scores represent the percentage of the maximum achievable score, ranging from 0 to 100%. According to the ELCWP scale, higher scores reflected a better methodological quality. It is important to point out that under the category “results analysis” only studies that performed a survival analysis could be evaluated. Thus, low global scores subsequently characterized studies that only investigated the association between an IAP family member and clinicopathological variables.

### 2.5. RNA-Seq Data

RNA-seq data from patients with lung adenocarcinoma (LUAD) or squamous cell carcinoma (LUSC) were extracted for each of the IAP family members (BIRC1-8) using the Kaplan–Meier Plotter (http://kmplot.com/analysis/index.php?p=service, accessed on 25 July 2021). This database includes the RNA-seq dataset from 513 LUAD and 501 LUSC specimens that originated from The Cancer Genome Atlas (TCGA) portal [60]. Kaplan–Meier survival curves were constructed for both OS and DFS. Survival curves were compared by using the log rank test and the HR with CI was estimated. To divide the patients according to the expression level of the respective IAP into high- and low-expression groups, the best performing threshold was selected to define the cut-off.

### 2.6. Statistical Analysis

The OR was calculated to measure the association between the investigated IAP family member and clinicopathological parameters. Clinicopathological parameters included sex, UICC stage, lymph node metastasis (N), histological differentiation (G), presence of distant metastases (M), smoking behavior, age, and tumor size (T). For comparability, some clinicopathological parameters were combined, including UICC stage I and II (early-stage) versus III and IV (advanced-stage), T1 and T2 versus T3 and T4, or well/moderate versus poor differentiation. The number of positive cases in each group of the analyzed clinicopathological parameters related to the total number of cases in the group was used for the analysis of the clinical parameters. HRs were used to display the significance of the expression levels of the IAP family members and the underlying results of the survival analysis. For patients with IAP overexpression, an HR > 1 indicated worse prognosis. In studies in which the HR and 95% CI were reported, we extracted them and used them to assess the pooled HR. In cases where the HR and 95% CI was missing or not available, we evaluated them by reading Kaplan–Meier survival curves with Engauge Digitizer software version 4.1 (http://digitizer.sourceforge.net/, accessed on 14 April 2020). For this purpose, we used the extracted data to reconstruct the HR and its variance by re-performing survival curve analysis (GraphPad Software, Inc., La Jolla, CA, USA), presuming that the number of censored cases was constant over the period of follow-up. By measuring inconsistency (I^2^) and using Cochrane’s Q test (Chi-squared test; Chi^2^), statistical heterogeneity was tested [59,61,62]. Assuming that the data to be analyzed consisted of different populations, ORs and HRs were pooled with 95% CI according to the method of DerSimonian and Laird (random effects model) [63]. Subgroup and one-way sensitivity analyses were used to test the stability of the meta-analysis. To perform the meta-analysis and to prepare the graphical results, we utilized Review Manager 5.0 (http://ims.cochrane.org/revman, accessed on 20 April 2020). Funnel plots were designed to visualize the risk of publication bias. For statistical testing of the funnel plot symmetry, Egger’s test and a rank correlation test were performed by JASP software [64]. In addition, the test of publication bias and the adjustment for publication bias were analyzed using selection models in JASP [64]. Quality scores between different subgroups were compared using non-parametric Mann–Whitney tests. A *p*-value < 0.05 indicated statistical significance.

## 3. Results

### 3.1. Study Selection and Characteristics

**Figure 2:**
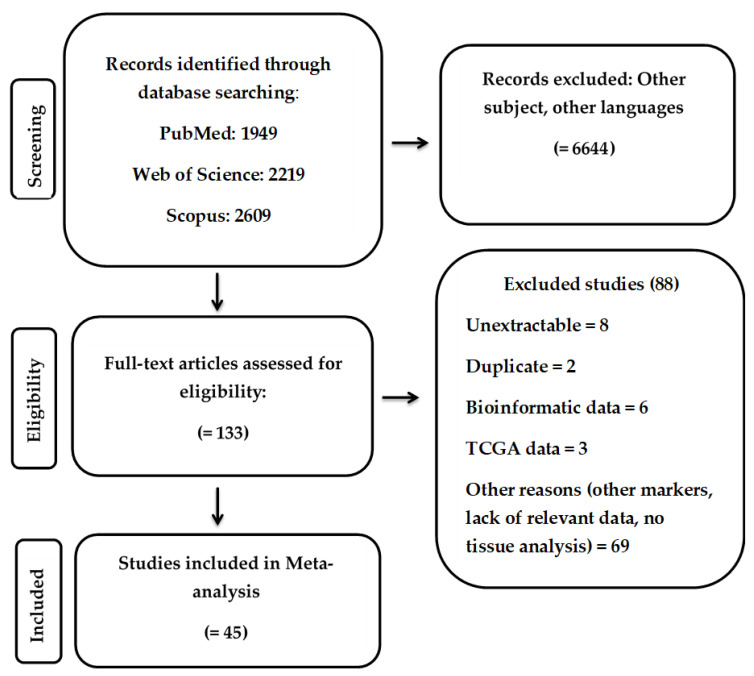
Flow chart summarizing the literature search and study selection.

In accordance with our defined search criteria, the electronic database search via PubMed, Web of Science, and Scopus revealed 1949, 2219, and 2609 articles, respectively (Figure 2). By meticulously reading the abstracts, we identified 133 studies that focused on the expression of the IAP family members survivin, XIAP, livin, and BRUCE. Of note, for other IAP family members our search strategy failed to identify eligible studies. After careful reading of the full text of the 133 studies, 8 articles were excluded because the analyzed data were not extractable for meta-analysis. Two studies were excluded because they were duplicates. Six studies analyzed bioinformatic data that were not relevant for meta-analysis, and were thus excluded. Three studies used TCGA data for survival analysis and 69 articles were excluded for other reasons (e.g., lack of relevant data, analyzed primarily other biomarkers, did not perform tissue analysis). Therefore, 45 eligible studies with 4428 patients (mean: 98.4; range: 32–373) and published between 1999 and 2017 [33,37,41,42,43,50,51,52,53,54,55,65,66,67,68,69,70,71,72,73,74,75,76,77,78,79,80,81,82,83,84,85,86,87,88,89,90,91,92,93,94,95,96,97,98] were included in our meta-analysis to assess the prognostic and clinicopathological significance of IAP family members as potential biomarkers in lung cancer. As illustrated in Table 1, 32 studies included patients from Asia, 11 studies patients from Europe, and 2 patients from North America. Seventeen studies included patients with UICC stage I–IV lung cancer, 12 studies investigated the expression of IAP family members in UICC stage I–III lung cancer, 4 studies assessed UICC stages III–IV, 3 studies analyzed UICC stages I and III lung cancer, and 2 studies investigated lung cancer patients of UICC stages I–II. Only 1 study assessed lung cancer patients with UICC stage II–III, and 3 studies did not provide information on the UICC stage. Regarding histological type, the majority of the studies (*n* = 41) enrolled only patients with NSCLC. Interestingly, 2 studies evaluated only patients with SCLC and 2 studies included patients with both tumor entities (NSCLC and SCLC). In addition, 25 studies included patients who underwent primary surgical resection and in 3 studies biopsy was performed prior to surgical resection. Three studies included patients treated with a combination of biopsy and chemotherapy and 12 studies included patients who underwent surgery and/or chemotherapy and/or radiation. Two studies did not provide any information on therapeutic modalities or the source of the samples. Expression of the IAPs was either detected by reverse transcriptase-polymerase chain reaction (RT-PCR) method (*n* = 7) or by immunohistochemistry (IHC; *n* = 36), whereas in 2 studies fluorescence in situ hybridization (FISH) was performed. Further methodological details of all included studies are summarized in Table S1.

Forty studies with a total of 4025 patients (mean: 105.5; range: 32–373) investigated the expression of the IAP family member survivin, 3 studies with 250 patients (mean: 83.3; range 34–144) investigated the expression of XIAP, and 2 studies with 156 as well as 2 studies comprising 118 patients analyzed the expression of livin and BRUCE, respectively (Table 1). For meta-analysis, 36 studies investigating survivin expression and 2 studies each that evaluated XIAP and livin expression, respectively, provided extractable survival data for a pooled survival analysis. Of note, the majority of these studies provided expression data in relation to overall survival. Survival data from 2 studies investigating BRUCE expression provided overall [97] and recurrence-free [53] survival, respectively, and thus could not be combined to estimate pooled HRs.

### 3.2. Study Quality

In order to evaluate the quality of the studies we included in our meta-analysis, we analyzed the study design, laboratory methodology, generalizability, and results analysis, and in addition calculated the global quality score of each study. We expressed the final global quality score as a percentage of the maximum achievable global score. Therefore, the mean global score of the included studies of our meta-analysis was 55.26% (range 32.21–63.16%) (Table 2). Of note, in the results analysis category only studies in which a survival analysis was performed could be evaluated. Therefore, five studies that did not provide survival data could not be assessed in this category and thus resulted in a low global quality score. Interestingly, as we compared the quality scores for study design, laboratory methodology, and generalizability of publications presenting survival data to those without survival data, a significantly higher value became evident for the study design category in the studies presenting survival data. As expected, studies that conducted a multivariate analysis achieved a significantly higher value for the results analysis category and thus, higher global quality scores. In addition, a significantly high value for laboratory methodology became evident when comparing studies that performed immunohistochemistry (IHC) with RNA-based (RT-PCR and FISH) studies. Moreover, there was no significant difference in the quality of studies from Asia or other countries.

### 3.3. Study Results and Meta-Analysis

**Figure 3:**
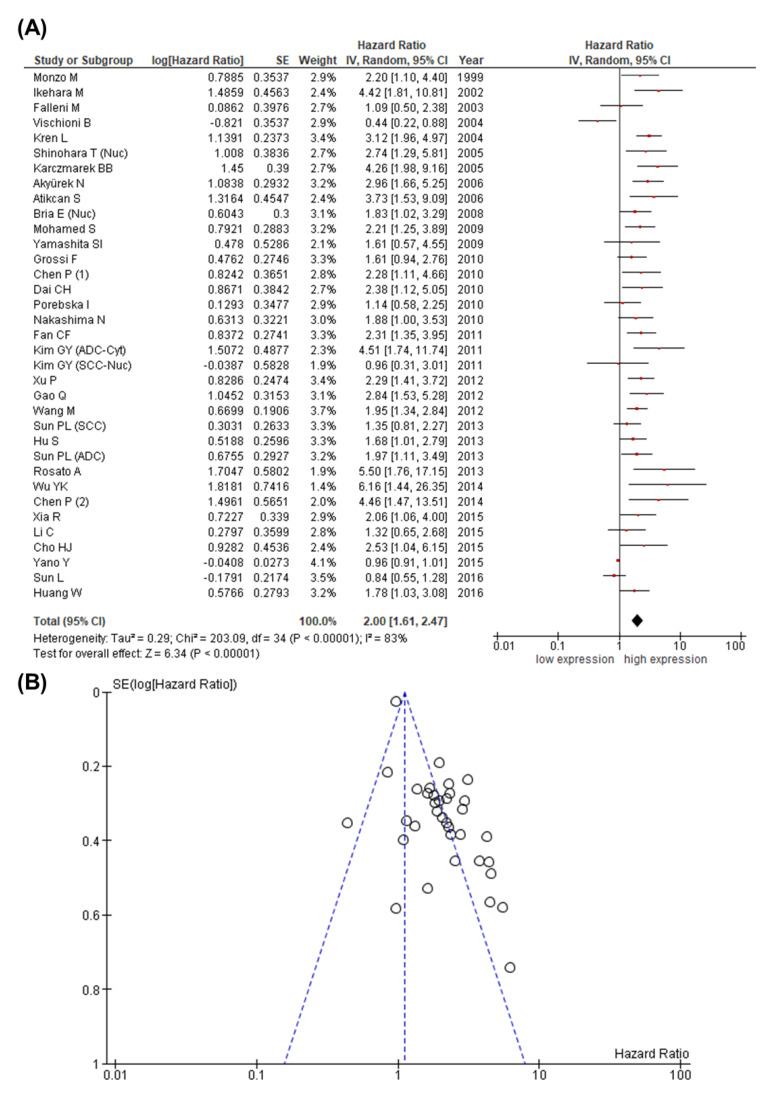
Meta-analysis comparing expression of survivin with OS in patients with lung cancer. (**A**) The forest plot reflects the individual and pooled HR with 95% CI. Heterogeneity was measured by the Cochrane Q test (Chi-squared test; Chi^2^) and inconsistency (I^2^). (**B**) The funnel plot demonstrates an asymmetric distribution. The *Y*-axis represents the standard error (SE), and the *x*-axis represents the study’s result.

Initially, we analyzed whether expression levels of the investigated IAP family members survivin, livin, XIAP, and BRUCE were associated with survival in patients with lung cancer. For comprehensibility, we described the results of the pooled HRs according to each investigated IAP family member. The majority of studies analyzed the association between the expression of the IAP family member survivin (*n* = 40, 4025 patients) either with overall survival (OS), disease-free survival (DFS), or disease-specific survival (DSS). In this group, the pooled HR of 33 studies showed that high survivin expression levels were associated with reduced overall survival in lung cancer patients (HR 2.00; 95% CI: 1.61–2.47; *p* < 0.00001) (Figure 3A). However, the Cochrane Q test (Chi^2^ = 203.09; *p* < 0.00001) and test of inconsistency (I^2^ = 83%) displayed significant heterogeneity. To perform one-way sensitivity analysis, we excluded step-by-step each included study and re-evaluated the pooled HR for the remaining studies (dataset not illustrated). However, heterogeneity remained consistent, implying that none of the studies was responsible for its occurrence. Next, we performed a subgroup analysis to identify possible determinants of heterogeneity. For this purpose, we investigated whether there were differences in outcomes related to detection method, HR estimate, global quality score, number of patients included, UICC stage, histological type, and country where the study was conducted (Table 3). Accordingly, heterogeneity was absent in studies using RT-PCR analysis for the detection of survivin or performing univariate survival analysis. In addition, heterogeneity became evident in studies that included patients with UICC stage IV. However, a discrimination depending on study quality, histological type, and country of origin failed to identify the source of heterogeneity. Next, we investigated whether the immunohistochemically analyzed subcellular localization of survivin could influence statistical heterogeneity and demonstrated that survival analysis based on the cytoplasmic expression of survivin was characterized by a moderate heterogeneity (Figure S1). In contrast, when nuclear or combined cytoplasmic/nuclear expression was evaluated or when studies did not define the subcellular localization, a substantial to considerable heterogeneity became evident. Furthermore, critical examination of the funnel plot displayed asymmetry (Figure 3B), therefore suggesting publication bias (Egger’s test: z = 3.063, *p* = 0.002; Begg test: Kendall’s tau = 0.061, *p* = 0.609). Hence, we applied a selection model using JASP that assessed and adjusted for publication bias [64]. Since the Chochrane Q test as well as inconsistency revealed heterogeneity, we first confirmed publication bias when applying the test that assumes heterogeneity (Chi^2^ 13.557, *p* < 0.001). Accordingly, we adjusted random effects estimates for publication bias, which confirmed a statistically significant relationship between survivin expression levels and OS in lung cancer patients (*p* = 0.043) (Figure S2A).

**Figure 4:**
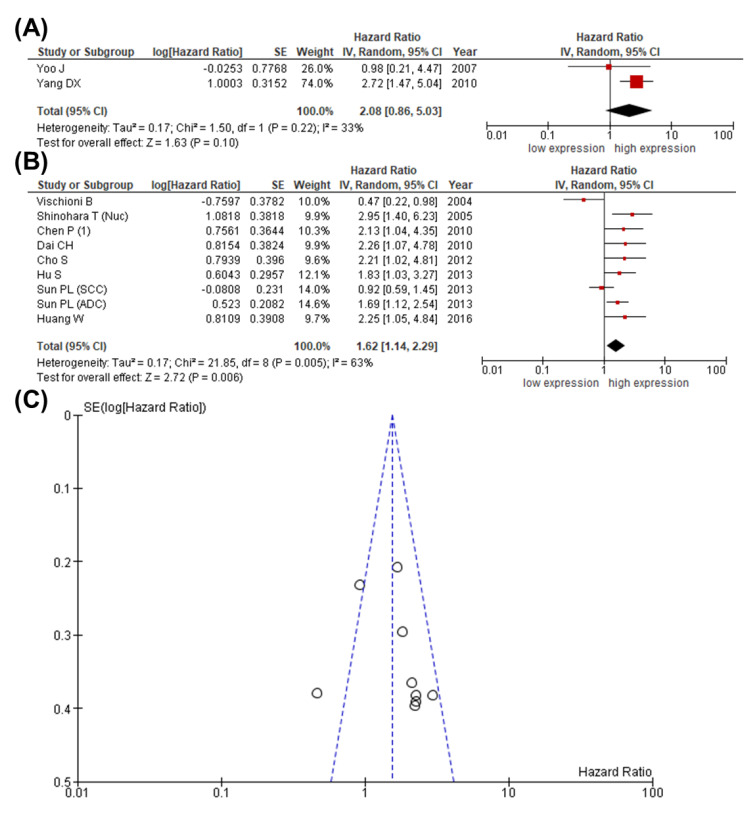
Meta-analysis comparing expression of survivin with DSS and DFS in patients with lung cancer. The forest plot reflects the individual and pooled HR with 95% CI to assess the association between survivin and (**A**) DSS or (**B**) DFS. Heterogeneity was quantified by the Cochrane Q test (Chi-squared test; Chi^2^) and inconsistency (I^2^). (**C**) The funnel plot for DFS demonstrates a symmetric distribution. The *Y*-axis represents the standard error (SE), and the *x*-axis represents the study’s result.

Whereas an association between expression of survivin and disease-specific survival (DSS) did not become evident (HR 2.08; 95% CI: 0.86–5.03; *p* = 0.10; I^2^ = 33%) (Figure 4A), the pooled HR of 8 studies that evaluated the association between survivin and DFS demonstrated the prognostic relevance of survivin expression in lung cancer patients, despite heterogeneity (HR 1.62; 95% CI: 1.14–2.29; *p* = 0.006; I^2^ = 63%) (Figure 4B).

Interestingly, the one-way sensitivity analysis no longer showed any heterogeneity after excluding the study by Vischioni [74]. Although the funnel plot (Figure 4C) combined with a statistical analysis for asymmetry (Egger’s test: z = 0.725, *p* = 0.468; Begg test: Kendall’s tau = 0.286, *p* = 0.399) did not reveal publication bias, we once again made use of the aforementioned selection model, in which the test for publication bias assuming heterogeneity suspected a publication bias (*p* < 0.001). Due to the observed heterogeneity, we adjusted random effects estimates for publication bias and confirmed the observation that high survivin expression was associated with a shorter DFS (*p* = 0.01) (Figure S2B).

**Figure 5:**
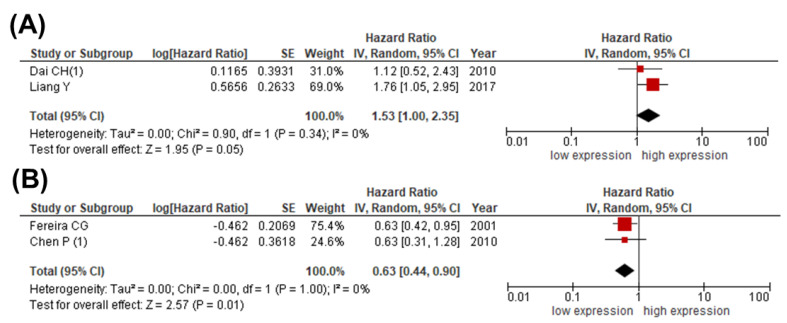
Meta-analysis comparing expression of (**A**) livin and (**B**) XIAP with OS in patients with lung cancer. The forest plot reflects the individual and pooled HR with 95% CI. Heterogeneity was measured by the Cochrane Q test (Chi-squared test; Chi^2^) and inconsistency (I^2^).

To investigate whether other IAP family members could serve as prognosticators in lung cancer patients, we next estimated the pooled HRs of studies providing XIAP or livin expression data for survival analysis. Whereas high livin expression was observed in patients with poor OS (HR 1.53; 95% CI: 1.00–2.35; *p* = 0.05; I^2^ = 0%), high XIAP was associated with favorable survival (HR 0.63; 95% CI: 0.44–0.90; *p* = 0.01; I^2^ = 0%) (Figure 5A,B).

To further elucidate the role of the IAP family members survivin, XIAP, livin, and BRUCE as biological markers, we investigated the association between their expression and clinicopathological parameters. Twenty-seven studies provided extractable data to compare survivin expression levels with clinicopathological parameters. Although heterogeneity became evident for some parameters, meta-analysis of these data using a random effects model revealed an association between high expression of survivin and advanced UICC stages (OR: 2.24; 95% CI: 1.56–3.21; *p* < 0.0001; I^2^ = 53%), T stage (OR: 1.57; 95% CI: 1.14–2.18; *p* = 0.006; I^2^ = 0%), poor histological differentiation (OR: 1.66; 95% CI: 1.20–2.29; *p* = 0.002; I^2^ = 37%), and the presence of lymph node metastasis (OR: 1.95; 95% CI: 1.36–2.78; *p* = 0.0003; I^2^ = 66%) (Table 4). However, meta-analysis of the expression of the remaining three members of the IAP family (XIAP, livin, and BRUCE) either showed no correlation with the investigated clinicopathological variables (data not shown) or was not performed as only a single study allowing data extraction (Tables S2–S4).

### 3.4. Validation Using the TCGA Dataset

To further compare the results from our meta-analysis including only expression data from the literature, we performed a survival analysis using RNA-seq datasets from TCGA comprising a total number of 1014 patients with lung cancer. Of note, a survival analysis including OS (Figure S3) and DFS (Figure S4) was performed for each IAP family member in LUAD and LUSC, separately. Whereas high expression levels of cIAP1/BIRC2 and survivin/BIRC5 were associated only with poor OS in LUAD, overexpression of cIAP2/BIRC3 correlated with a shorter OS in both LUAD and LUSC. Moreover, increased NAIP/BIRC1 levels were detectable in LUSC patients with a shorter OS. In contrast, high NAIP/BIRC1 or livin/BIRC7 and Ts-IAP/BIRC8 expression were associated with a more favorable OS in LUAD and LUSC, respectively. However, when correlating the expression levels with DFS, high expression of cIAP1/BIRC2 and XIAP/BIRC5 were associated with a shorter DFS in LUAD and LUSCD, respectively. This was the opposite for NAIP/BIRC1 and Ts-IAP/BIRC8 in LUAD.

Next, we combined the HRs obtained for each IAP family member from LUAD and LUSC and compared the summarized HR (Table S5) with the pooled HR obtained from our meta-analysis. Accordingly, only for DFS did the summarized HR from both our literature search (HR 1.62; 95% CI: 1.14–2.29; *p* = 0.006) and Ref-seq dataset (HR 1.58; 95% CI: 1.13–2.20; *p* = 0.007) demonstrate an association for survivin/BIRC5 with recurrent disease.

## 4. Discussion

Despite enormous advances in therapeutic modalities, lung cancer remains the leading cause of cancer-related deaths worldwide [1]. Although the clinical outcome for a subset of patients using targeted therapy and immunoregulatory molecules has improved, the five-year survival rate is still less than 20% [2,9,31]. The inhibitors of apoptosis protein (IAP) family represent a heterogeneous group of anti-apoptotic proteins that have been shown to be overexpressed in a variety of malignant diseases [33,34,35,36,37]. Hence, over the past decade, various members of the IAP family have attracted considerable interest as treatment targets in cancer therapy and have been demonstrated to correlate with poor prognosis in several studies investigating different tumor entities [34,35,36,37,42,50,51,52,53,54,55]. However, some of the published studies already reported contradictory outcome data related to a single cancer entity. Accordingly, it is of utmost importance to synthesize these data in a meta-analysis to elucidate potential correlations between IAP family members and clinicopathological parameters as well as prognosis in cancer patients.

For this purpose, we conducted a meta-analysis of 45 eligible studies that aimed to investigate for the first time an association between all IAP family members and clinicopathological parameters or survival outcomes in patients with lung cancer. Of all these included studies, the majority (*n* = 40, 4025 patients) investigated the expression of survivin in lung cancer, of which 33, 8, and 2 investigated OS, DFS, and DSS, respectively. It should be mentioned that some studies examined more than one IAP family member or survival outcome in the same study and thus were included in each analyzed IAP group or outcome separately [43,52,69,84,94]. Despite heterogeneity, we found that high expression of survivin correlated with poor overall OS and DFS, but not with disease-specific survival. The latter might be due to the low number of studies that investigated this outcome. In addition, due to the high number of studies (*n* = 33) that examined the correlation between the expression of survivin and OS, we were able to perform a subgroup analysis to determine whether survivin subcellular localization correlated with survival. Interestingly, high cytoplasmic as well as nuclear survivin expression levels were associated with poor overall survival. Moreover, a meta-analysis of studies that analyzed survivin expression in both subcellular compartments (nuclear and cytoplasmic) also demonstrated poor prognosis of elevated survivin expression. Our result regarding cytoplasmic survivin expression is in line with a previous meta-analysis that investigated the correlation between survivin expression and prognosis in hepatocellular carcinoma [99]. However, in the meta-analysis by Fan [100] that investigated the association between survivin and OS in NSCLC, positive nuclear survivin expression did not turn out to serve as a prognostic factor for overall survival, which is contradictory to our result. This difference might be due to the low number of studies included in this study compared to our meta-analysis. For the 33 studies that performed OS analysis and the 8 studies that analyzed DFS with respect to survivin expression, we found considerable to substantial heterogeneity after calculating the pooled HR. To detect determinants of heterogeneity, we performed one-way sensitivity and subgroup analyses. Whereas heterogeneity remained consistent for OS when performing one-way sensitivity analysis, exclusion of the study published by Vischioni [74] adjusted the problem of heterogeneity for DFS. However, one main cause of heterogeneity might be explainable by the inclusion of studies using different detection methods, such as FISH, RT-PCR, and IHC. Importantly, none of the detection methods was either validated or standardized. In addition, studies in which expression of survivin was detected by IHC sometimes used different antibodies from different species and used heterogeneous cut-off values to define positive or high protein expression. Moreover, some studies included patients of all UICC stages, whereas others excluded UICC IV patients or did not specify the UICC stages included.

In addition, we would like to emphasize further limitations of our meta-analysis. Since we had to extract Kaplan–Meier survival data from some publications and therefore had to assume that the number of censored cases remained constant during follow-up, the re-calculated HRs from these survival curves might be less accurate. Of note, a relevant bias might be introduced by the retrospective design of the included studies. Moreover, our meta-analysis contained publication bias that might be explainable by the inclusion of studies written only in English and our search strategy that did not take grey literature into account. Therefore, we applied a selection model and conducted a publication bias-adjusted meta-analysis. In this context, we have to admit that these models have their limitations when the number of studies is small.

With regard to the other IAP family members, XIAP, livin, and BRUCE, a meta-analysis of survival outcomes was only conducted for XIAP and livin, as the 2 eligible studies that analyzed BRUCE investigated different outcomes. The pooled HR of the 2 studies that consecutively examined the association between expression levels XIAP and livin revealed that high livin and low XIAP expression correlated with poor survival. Since pooled HRs were synthesized only by 2 studies for each IAP, these results have to be interpreted with caution. However, the result of our meta-analysis for livin is the first report that investigates the correlation between livin expression levels and overall survival.

To further validate the results from our meta-analysis, we made use of the publicly available TCGA datasets from lung cancer patients and performed for each single IAP family member a Kaplan–Meier analysis for OS and DFS, respectively. By using this approach, we could only confirm the results from our meta-analysis that demonstrated the association between high survivin expression and shorter DFS. Since the TCGA data originate from RNA-sequencing and the expression analysis of our meta-analysis was performed by other methods such as RT-PCR, FISH, and immunohistochemistry, the comparison between the TCGA-based survival analysis and the results from our literature-based meta-analysis have to be interpreted with caution. Nonetheless, more studies are needed to verify our results regarding lung cancer. Additionally, in our meta-analysis we analyzed the correlation between expression levels of the respective IAP family member and clinicopathological parameters in patients with lung cancer. Our results demonstrated that the expression of survivin correlates with advanced UICC stage, differentiation, advanced T stage, and the presence of lymph node metastasis. However, heterogeneity became evident when comparing survivin overexpression with UICC stages as well as the presence of lymph node metastasis. In this context, dissimilar extensiveness of lymphadenectomy during surgery among the eligible studies, the application of different multimodal therapy regimens (radiotherapy and/or chemotherapy and/or surgery), or the use of different classification systems might have caused heterogeneity for nodal status and UICC stage. Of note, none of the remaining 3 members (XIAP, livin, and BRUCE) demonstrated a correlation with the investigated clinicopathological data, which can be explained by the low number of eligible studies. Thus, more studies are needed that investigate the clinical impact of these IAPs in lung cancer.

To date, various small-molecule inhibitors targeting IAPs have been investigated. Of these, SMAC mimetics (primary IAP antagonists) play a major role as synthetic mimics of the endogenous second mitochondria-derived caspase activator/direct IAP binding protein with low isoelectric point (SMAC/DIABLO) [101,102]. Endogenous SMAC/DIABLO exerts its inhibitory effect on IAPs by binding to the BIR domain, competitively inhibiting their binding with effector caspases 9, 3, and 7 [103]. In a previous study, novel SMAC mimetic LCL161 has been shown to increase paclitaxel-induced apoptosis by degrading cIAP1 and cIAP2 in NSCLC [104]. Specifically with regard to survivin, the most extensively studied IAP family member in lung cancer to date, several previous and ongoing clinical trials have been and are being conducted using survivin as a target for cancer therapy. YM155 (sepantronium bromide) was the first small-molecule inhibitor discovered to target the expression of survivin. YM155 inhibits survivin promoter-driven expression without interfering with the expression of other anti-apoptotic proteins. However, it is well tolerated, with a maximum dose of 4.8 mg/m^2^ [105], and was reported to have moderate efficacy as single agent for some tumors [106]. However, in previous phase II trials of YM155 as a single therapy or in combination with carboplatin and paclitaxel for patients with NSCLC, no improvement of response rates was observed [107,108]. Recently, a phase I study evaluating the safety and pharmacokinetics of YM155 in combination with erlotinib in patients with EGFR TKI refractory advanced NSCLC demonstrated a favorable safety profile and moderate clinical efficacy when YM155 was administered up to 8.0 mg/m^2^/day every three weeks [109]. A novel small-molecule transcriptional repressor of survivin, EM-1421 (terameprocol), which is being tested in ongoing clinical trials, was demonstrated in clonogenic survival assays to induce radiosensitization in NSCLC cells [110].

Another investigated group of cancer therapeutics that inhibit survivin are antisense oligonucleotides, including LY2181308 and SPC3042/EZN-3042. In clinical studies, these oligonucleotides displayed mixed results. LY2181308 is a 2-*O*-methoxymethyl-modified single-strand antisense oligonucleotide that inhibits survivin mRNA to decrease survivin expression [111], whereas SPC3042 is a locked antisense oligonucleotide that targets the stop codon of the open reading frame in exon 4 of the survivin transcript [112].

Unfortunately, the results of clinical trials using small-molecule survivin inhibitors have not yet met the initially high expectations. However, another promising attempt to target survivin might be an immunotherapeutic approach including T cell-based or dendritic cell (DC)-based vaccines, which were well tolerated in the first phase I and IIa studies [113,114]. Currently, a study in NSCLC investigating a combination therapy with nivolumab and autologous DCs that are also pulsed with survivin is still recruiting patients (Clinical Trials identifier: NCT04199559).

## 5. Conclusions

In conclusion, our meta-analysis shows that high expression of certain members of the IAP family are associated with poor overall survival and disease recurrence in patients with lung cancer. Whereas more data are needed to reveal the prognostic relevance of distinct IAP family members in lung cancer, survivin, as the most extensively studied member of the IAP family, displays high impact as a biomarker independently of its subcellular localization. Therefore, survivin could be a promising therapeutic target in the development of innovative multimodal therapies for lung cancer.

## Figures and Tables

**Table 1:** Clinical characteristic of included studies.

First Author	Year	Country	Stage	Histology	Study Period	No. of Patients	Age (* Mean/^#^ Median)	Marker	Sex (Female/Male)	Therapy/Source of Samples	* Mean/^#^ Median Follow up (Months)
Yano, Y. [65]	2015	Japan	I–IV	SCLC	2003–2012	32	68 ^#^ (45–82)	SVV	4/28	Surgery (*n* = 16)/Surgery, Biopsy	NA
Karczmarek-Borowska, B. [51]	2005	Poland	II–III	NSCLC	1993–1997	60	57 * (38–69)	SVV	6/54	Surgery + adj. chemotherapy	22 ^#^ (5–60)
Rosato, A. [33]	2013	Italy	I–II I–II	NSCLC SCLC	2002–2009 2002–2009	65 35	NA NA	SVV SVV	17/48 12/23	Surgery NA/Biopsy	NA
Chen, P. (1) [37]	2010	China	III–IV	NSCLC	2002–2006	72	59 ^#^ (40–77)	SVV XIAP	21/51	Chemotherapy/Biopsy	NA
Dai, C.H. [52]	2010	China	I–III	NSCLC	2003–2005	66	60 ^#^ (34–78)	SVV Livin	20/46	Surgery + adj. chemotherapy; in 5 cases + radiation/Surgery	52 ^#^
Bria, E. [42]	2008	Italy	I–III	NSCLC	1995–2001	116	60 ^#^	SVV	15/101	Surgery + adj. chemotherapy (*n* = 45), radiochemotherapy (*n* = 30)	20 ^#^ (10–129)
Yoo, J. [67]	2007	South Korea	I–III	NSCLC	NA	219	67(19–89)	SVV	51/168	Surgery	38.9 ^#^ (1.6–117.8)
Monzó, M. [68]	1999	Spain	I–III	NSCLC	1995–1996	83	65 ^#^ (26–77)	SVV	5/78	Surgery	18 ^#^ (2–31)
Sun, P.L. [69]	2013	South Korea	I–IV	NSCLC	2003–2009	373	65 ^#^ (21–84)	SVV	115/258	Surgery + adj. chemotherapy	Range (0–80)
Xu, P. [70]	2012	China	I–III	NSCLC	2006–2007	97	60 ^ND^	SVV	22/75	Surgery	Range (4–58)
Chen, P. (2) [66]	2014	China	I–IV	SCLC	2000–2007	45	53 ^#^ (30–76)	SVV	8/37	Surgery + adj. chemotherapy (*n* = 8) or radiochemotherapy (*n* = 37)	11 ^#^
Gao, Q. [71]	2012	China	I–IV	NSCLC	2001–2005	62	57.8 * (35–78)	SVV	18/44	Surgery	Range (3–120)
Hu, S. [43]	2013	China	I–III	NSCLC	2004–2006	256	57.7 *	SVV	80/176	Surgery + adj. chemotherapy (*n* = 217), radiotherapy (*n* = 92)	64 ^#^
Mohamed, S. [72]	2009	Japan	NA	NSCLC	1990–1996	78	62.8 *	SVV	20/58	Surgery	NA
Shinohara, E.T. [41]	2005	USA	I–II	NSCLC	1996–2002	144	67 ^#^ (34–92)	SVV	50/94	Surgery	NA
Cho, S. [73]	2012	South Korea	I	NSCLC	2003–2006	110	62.3 * (41–79)	SVV	26/84	Surgery	55 ^#^ (2.3−87.9)
Vischioni, B. [74]	2004	Netherlands	III–IV	NSCLC	1993–1999	53	56 * (29–75)	SVV	20/33	Neoadj. chemotherapy, surgery, or radiotherapy (*n* = 32), palliative chemotherapy (*n* = 21)	76 ^#^
Wang, M. [75]	2012	China	III	NSCLC	2002–2004	210	59.8 * (35–76)	SVV	80/130	Surgery	NA
Wu, Y.K. [76]	2014	Taiwan	III–IV	NSCLC	2004–2009	48	59.4 * (36–83)	SVV	16/32	Biopsy, chemotherapy	20.4 ^#^ (3.4–59)
Yamashita, S.I. [77]	2009	Japan	NA	NSCLC	1997–2003	47	NA	SVV	14/33	Surgery	64.8 ^#^ (14.4–100.8)
Akyürek, N. [50]	2006	Turkey	I–IV	NSCLC	1994–2001	78	63 ^#^ (39–78)	SVV	6/72	Surgery (*n* = 27), biopsy (*n* = 51)	18 ^#^ (1–80)
Cho, H.J. [78]	2015	South Korea	III	NSCLC	2000–2005	53	57.6 *	SVV	10/43	Neoadj. radiochemotherapy, Surgery adj. radio- (*n* = 25) or radiochemotherapy (*n* = 2)	NA
Falleni, M. [79]	2003	Italy	I	NSCLC	NA	83	63.4 * (43–74)	SVV	4/79	Surgery	54 ^#^ (7–94)
Hirano, H. [80]	2014	Japan	I	NSCLC	2007–2010	44	65. 3 ^ND^ ± 1.5	SVV	8/36	Surgery	38.3 ^ND^ (5.2–58.9)
Kren, L. [54]	2004	Czech Republic	I–III	NSCLC	1983–1994	102	NA	SVV	45/57	Surgery	NA
Porebska, I. [81]	2010	Poland	I–IV	NSCLC	NA	74	60.5 * (43–77)	SVV	25/49	Surgery, neoadj. therapy (*n* = 22), palliative therapy	NA
Nakashima, N. [82]	2010	Japan	I–III	NSCLC	2001–2004	122	NA	SVV	NA	Surgery	NA
Atikcan, S. [83]	2006	Turkey	I–III	NSCLC	2000–2003	58	57.29 * (40–76)	SVV	0/58	Surgery, adj. chemotherapy (*n* = 9), radiotherapy (*n* = 13), radiochemotherapy (*n* = 5)	NA
Huang, W. [84]	2016	China	III–IV	NSCLC	2006–2011	61	56 ^#^ (32–74)	SVV	15/46	Chemotherapy, biopsy	7 ^#^ (2–29)
Kim, G.Y. [85]	2011	South Korea	I–IV	NSCLC ADC SCC	1985–2005	244 93 151	62 * (35-81) NA NA	SVV	55/189 40/53 15/136	Surgery	NA
Yang, D.X. [86]	2010	China	I–IV	NSCLC	2002–2004	60	53.5 ^#^ (37–71)	SVV	20/40	Surgery	NA
Chen, Y.Q. [87]	2009	China	I–III	NSCLC	2005–2007	120	61 * (42–76)	SVV	26/94	Surgery, adj. chemotherapy	NA
Ikehara, M. [88]	2002	Japan	I–IV	NSCLC	1992–1999	79	64 ^#^ (26–83)	SVV	46/33	Surgery	NA
Fan, C.F. [89]	2011	China	I–IV	NSCLC	1998–2005	76	57.1 * (26–78)	SVV	30/46	Surgery	45.6 ^#^ (3–111)
Yu, S. [90]	2014	China	I–IV	NSCLC	2006–2013	32	60.1 * (43–82)	SVV	9/23	Surgery	NA
Li, C. [91]	2015	China	I–IV	NSCLC	NA	75	59 ^#^ (20–84)	SVV	36/39	Surgery	NA
Grossi, F. [92]	2010	Italy	III	NSCLC	1985–1997	87	62 ^#^ (35–74)	SVV	16/71	Surgery, adj. radiotherapy (*n* = 44)	140. 4 ^#^ (61.2–214.8)
Wang, X.Y. [93]	2005	China	I–IV	NSCLC and SCLC	NA	54	60 ^#^ (33–78)	SVV	17/37	NA	NA
Xia, R. [94]	2015	China	I–IV	NSCLC	2004–2008	104	NA	SVV	26/78	Surgery	40 ^#^ (8–89)
Liang, Y. [55]	2017	China	I–IV	NSCLC	2004–2009	90	NA	Livin	41/49	Surgery	NA
Ferreira, C.G. [95]	2001	Netherlands	I–III	NSCLC	1988–1995	144	65 ^ND^	XIAP	24/120	Surgery	104 ^#^
Hofmann, H.S. [96]	2002	Germany	I–IV	NSCLC	1999–2000	34	NA	XIAP	NA	Surgery	NA
Dong, X. [53]	2013	Canada	I–IV	NSCLC	2005–2006	78	69.09 * ± 9.52	BIRC6	44/34	Surgery	NA
Gharabaghi, M.A. [97]	2016	Iran	NA	NSCLC	2006–2014	40	NA	BIRC6	17/23	Surgery	NA
Sun, L. [98]	2016	China	I–IV	NSCLC	NA	165	NA	SVV	45/120	NA	NA

adj.: adjuvant; neoadj.: neoadjuvant; SVV: survivin, NSCLC: non-small-cell lung cancer; SCLC: small-cell lung cancer; XIAP: X-linked inhibitor of apoptosis protein; BIRC6: baculoviral IAP repeat containing 6 (BRUCE); NA: not available; ND: not defined; *: mean; #: median.

**Table 2:** Study quality assessment according to the ELCWP Scale.

	No. of Studies	Design	Laboratory Methodology	Generalizability	Results Analysis	Global Score (%)
All studies	45	6.00	6.00	4	6	55.26
Survival data	40	7	6	4	6	60.53
No survival data	5	5	4	4	NA	34.21
*p*		<0.001	0.26	0.37	NA	0.001
UV	13	6	6	4	4	55.26
MV	27	7	5	5	6	63.16
*p*		0.15	0.43	0.07	<0.001	0.009
IHC	36	7	6	4	6	60.53
RNA-based	9	6	4	4	6	50
*p*		0.11	0.002	0.24	0.97	0.01
Asian	32	6	6	4	6	55.26
Other regions	13	7	5	6	6	60.53
*p*		0.39	0.92	0.88	0.73	0.39

ELCWP: European Lung Cancer Working Party; UV: univariate; MV: multivariate; IHC: immunohistochemistry; NA: not available.

**Table 3:** Subgroup analysis of summarized hazard ratios reflecting the relationship between survivin/BIRC5 and overall survival in lung cancer.

				Pooled Data (Random)			Test for Heterogeneity	
Subgroup	No. of Studies	Cases	HR	95% CI	*p*-Value	Chi^2^	*p*-Value	I^2^ (%)
Method								
IHC	26	2892	1.92	1.52–2.42	<0.00001	170.44	<0.00001	84
PCR	6	451	2.09	1.45–3.00	<0.0001	5.89	0.32	15
FISH	1	60	4.26	1.98–9.16	NA	NA	NA	NA
Survival analysis								
Kaplan–Meier	9	758	2.21	1.69–2.88	<0.00001	13.7	0.09	42
UV	4	353	1.54	1.10–2.16	0.01	1.44	0.7	0
MV	21	2292	2.01	1.52–2.65	<0.00001	130.19	<0.00001	84
Global Quality Score								
≥57.89	19	2013	2.14	1.75–2.63	<0.00001	38.56	0.005	51
<57.89	14	1390	1.76	1.30–2.37	0.0002	64.48	<0.00001	78
Cases (N)								
≥78	18	2521	1.99	1.64	<0.00001	38.4	0.005	51
<78	15	882	1.96	1.36–2.81	0.0003	83.24	<0.00001	83
UICC								
I-IV	12	1407	1.83	1.32–2.54	0.0003	75.84	<0.00001	83
I-III	8	900	2.03	1.60–2.58	<0.00001	12.9	0.12	38
w/o IV	15	1602	2.21	1.87–2.62	<0.00001	15.89	0.32	12
NA	2	125	2.05	1.25–3.37	0.004	0.27	0.6	0
Histological Type								
NSCLC	31	3326	1.99	1.68–2.36	<0.00001	70.97	<0.0001	55
SCLC	2	77	1.87	0.42–8.30	0.41	7.38	0.007	86
Country								
Asian	23	2501	2.04	1.60–2.62	<0.00001	141.66	<0.00001	83
Caucasian	10	902	1.87	1.23–2.84	0.003	34.72	<0.0001	74

**Table 4:** Association between survivin/BIRC5 and clinicopathological variables.

			Pooled Data (Random)			Test for Heterogeneity		
Clinicopathological Variable	No. of Studies	Cases	OR	95% CI	*p*-Value	Chi^2^	*p*-Value	I^2^ (%)
Sex (female/male)	22	2387	0.92	0.70–1.20	0.52	32.63	0.05	36
UICC stage (I + II/III + IV)	18	2160	2.24	1.56–3.21	<0.0001	36.09	0.004	53
T stage (T1 + 2/T3 + 4)	12	1515	1.57	1.14–2.18	0.006	7.22	0.78	0
Differentiation (well + moderate/poor)	17	1778	1.66	1.20–2.29	0.002	25.21	0.07	37
Lymph node metastasis	23	2687	1.95	1.36–2.78	0.0003	64.26	<0.00001	66
Distant metastasis	5	484	0.84	0.47–1.51	0.56	4.14	0.39	3
Smoking	8	969	1.11	0.84–1.46	0.73	4.82	0.68	0
Age	16	2016	0.99	0.78–1.25	0.91	19.3	0.2	22
Tumor size	7	859	0.98	0.59–1.61	0.93	13.21	0.04	55

## Data Availability

The datasets used and/or analyzed during the current study are available from the corresponding author on reasonable request.

## Supplementary Materials

The following are available online at https://www.mdpi.com/article/10.3390/cancers13164098/s1, Figure S1: Meta-analysis comparing the subcellular expression of survivin with OS in patients with lung cancer. The forest plot reflects the individual and pooled HR with 95% CI. Heterogeneity was quantified by the Cochrane Q test (Chi-squared test; Chi^2^) and inconsistency (I^2^). Figure S2: Comparison of effect size estimates of the adjusted/unadjusted random effects model for the association of survivin expression and (A) OS or (B) DFS. After adjusting for publication bias, the estimated effect sizes are still statistically significant. Mean estimates (µ) correspond to the log (hazard ratio), Figure S3: Kaplan–Meier curves for overall survival (OS) in lung cancer patients depending on the IAP/BIRC family member. The TCGA dataset was used to analyze the expression of (A, B) NAIP/BIRC1, (C, D) cIAP1/BIRC2, (E, F) cIAP2/BIRC3, (G, H) XIAP/BIRC4, (I, J) survivin/BIRC5, (K, L) BRUCE/BIRC6, (M, N) livin/BIRC7, and (O, P) Ts-IAP/BIRC8 in (A, C, E, G, I, K, M, O) lung adenocarcinoma (LUAD) or (B, D, F, H, J, L, N, P) lung squamous cell carcinoma (LUSC), Figure S4: Kaplan–Meier curves for disease-free survival (DFS) in lung cancer patients depending on the IAP/BIRC family member. The TCGA dataset was used to analyze the expression of (A, B) NAIP/BIRC1, (C, D) cIAP1/BIRC2, (E, F) cIAP2/BIRC3, (G, H) XIAP/BIRC4, (I, J) survivin/BIRC5, (K, L) BRUCE/BIRC6, (M, N) livin/BIRC7, and (O, P) Ts-IAP/BIRC8 in (A, C, E, G, I, K, M, O) lung adenocarcinoma (LUAD) or (B, D, F, H, J, L, N, P) lung squamous cell carcinoma (LUSC), Table S1: Methodological characteristics of included studies, Table S2: Association between XIAP/BIRC4 and clinicopathological variables, Table S3: Association between livin/BIRC7 and clinicopathological variables, Table S4: Association between BRUCE/BIRC6 and clinicopathological variables, Table S5: HR and 95% CI according to the expression of IAP/BIRC family members in LUAD, LUSC, and the combined histological subtypes (LUAD + LUSC) using TCGA datasets.

## References

[1] Bray F., Ferlay J., Soerjomataram I., Siegel R.L., Torre L.A., Jemal A. (2018). Global cancer statistics 2018: GLOBOCAN estimates of incidence and mortality worldwide for 36 cancers in 185 countries. CA Cancer J. Clin..

[2] Travis W.D., Brambilla E., Nicholson A.G., Yatabe Y., Austin J.H.M., Beasley M.B., Chirieac L.R., Dacic S., Duhig E., Flieder D.B. (2015). The 2015 World Health Organization classification of lung tumors: Impact of genetic, clinical and radiologic advances since the 2004 classification. J. Thorac. Oncol..

[3] Goldstraw P., Chansky K., Crowley J., Rami-Porta R., Asamura H., Eberhardt W.E., Nicholson A.G., Groome P., Mitchell A., Bolejack V. (2016). The IASLC lung cancer staging project: Proposals for revision of the TNM stage groupings in the forthcoming (eighth) edition of the TNM classification for lung cancer. J. Thorac. Oncol..

[4] Nicholson A.G., Chansky K., Crowley J., Beyruti R., Kubota K., Turrisi A., Eberhardt W.E., van Meerbeeck J., Rami-Porta R. (2016). The international association for the study of lung cancer lung cancer staging project: Proposals for the revision of the clinical and pathologic staging of small cell lung cancer in the forthcoming eighth edition of the TNM classification for lung cancer. J. Thorac. Oncol..

[5] Nagasaka M., Gadgeel S.M. (2018). Role of chemotherapy and targeted therapy in early-stage non-small cell lung cancer. Expert Rev. Anticancer Ther..

[6] Duma N., Santana-Davila R., Molina J.R. (2019). Non-small cell lung cancer: Epidemiology, screening, diagnosis, and treatment. Mayo Clin. Proc..

[7] Dela Cruz C.S., Tanoue L.T., Matthay R.A. (2011). Lung cancer: Epidemiology, etiology, and prevention. Clin. Chest Med..

[8] Asamura H., Aokage K., Yotsukura M. (2017). Wedge resection versus anatomic resection: Extent of surgical resection for stage I and II lung cancer. Am. Soc. Clin. Oncol. Educ. Book.

[9] Osmani L., Askin F., Gabrielson E., Li Q.K. (2018). Current WHO guidelines and the critical role of immunohistochemical markers in the subclassification of non-small cell lung carcinoma (NSCLC): Moving from targeted therapy to immunotherapy. Semin. Cancer Biol..

[10] Rusch V.W., Crowley J., Giroux D.J., Goldstraw P., Im J.G., Tsuboi M., Tsuchiya R., Vansteenkiste J. (2007). The IASLC lung cancer staging project: Proposals for the revision of the N descriptors in the forthcoming seventh edition of the TNM classification for lung cancer. J. Thorac. Oncol..

[11] Tsao M.S., Sakurada A., Cutz J.C., Zhu C.Q., Kamel-Reid S., Squire J., Lorimer I., Zhang T., Liu N., Daneshmand M. (2005). Erlotinib in lung cancer—Molecular and clinical predictors of outcome. N. Engl. J. Med..

[12] Soda M., Choi Y.L., Enomoto M., Takada S., Yamashita Y., Ishikawa S., Fujiwara S., Watanabe H., Kurashina K., Hatanaka H. (2007). Identification of the transforming EML4-ALK fusion gene in non-small-cell lung cancer. Nature.

[13] Le X., Nilsson M., Goldman J., Reck M., Nakagawa K., Kato T., Ares L.P., Frimodt-Moller B., Wolff K., Visseren-Grul C. (2021). Dual EGFR-VEGF pathway inhibition: A promising strategy for patients with EGFR-mutant NSCLC. J. Thorac. Oncol..

[14] Rosell R., Carcereny E., Gervais R., Vergnenegre A., Massuti B., Felip E., Palmero R., Garcia-Gomez R., Pallares C., Sanchez J.M. (2012). Erlotinib versus standard chemotherapy as first-line treatment for European patients with advanced EGFR mutation-positive non-small-cell lung cancer (EURTAC): A multicentre, open-label, randomised phase 3 trial. Lancet Oncol..

[15] Jänne P.A., Yang J.C., Kim D.W., Planchard D., Ohe Y., Ramalingam S.S., Ahn M.J., Kim S.W., Su W.C., Horn L. (2015). AZD9291 in EGFR inhibitor-resistant non-small-cell lung cancer. N. Engl. J. Med..

[16] Sharma S.V., Bell D.W., Settleman J., Haber D.A. (2007). Epidermal growth factor receptor mutations in lung cancer. Nat. Rev. Cancer.

[17] Sequist L.V., Yang J.C., Yamamoto N., O’Byrne K., Hirsh V., Mok T., Geater S.L., Orlov S., Tsai C.M., Boyer M. (2013). Phase III study of afatinib or cisplatin plus pemetrexed in patients with metastatic lung adenocarcinoma with EGFR mutations. J. Clin. Oncol..

[18] Mok T.S., Wu Y.L., Thongprasert S., Yang C.H., Chu D.T., Saijo N., Sunpaweravong P., Han B., Margono B., Ichinose Y. (2009). Gefitinib or carboplatin-paclitaxel in pulmonary adenocarcinoma. N. Engl. J. Med..

[19] Ribeiro T.B., Buss L., Wayant C., Nobre M.R.C. (2020). Comparison of FDA accelerated vs. regular pathway approvals for lung cancer treatments between 2006 and 2018. PLoS ONE.

[20] Lin J.J., Cardarella S., Lydon C.A., Dahlberg S.E., Jackman D.M., Jänne P.A., Johnson B.E. (2016). Five-year survival in EGFR-mutant metastatic lung adenocarcinoma treated with EGFR-TKIs. J. Thorac. Oncol..

[21] Facchinetti F., Marabelle A., Rossi G., Soria J.C., Besse B., Tiseo M. (2016). Moving immune checkpoint blockade in thoracic tumors beyond NSCLC. J. Thorac. Oncol..

[22] Brahmer J.R., Tykodi S.S., Chow L.Q., Hwu W.J., Topalian S.L., Hwu P., Drake C.G., Camacho L.H., Kauh J., Odunsi K. (2012). Safety and activity of anti-PD-L1 antibody in patients with advanced cancer. N. Engl. J. Med..

[23] Sławiński G., Wrona A., Dąbrowska-Kugacka A., Raczak G., Lewicka E. (2020). Immune checkpoint inhibitors and cardiac toxicity in patients treated for non-small lung cancer: A review. Int. J. Mol. Sci..

[24] Sheppard K.A., Fitz L.J., Lee J.M., Benander C., George J.A., Wooters J., Qiu Y., Jussif J.M., Carter L.L., Wood C.R. (2004). PD-1 inhibits T-cell receptor induced phosphorylation of the ZAP70/CD3zeta signalosome and downstream signaling to PKCtheta. FEBS Lett..

[25] Pardoll D.M. (2012). The blockade of immune checkpoints in cancer immunotherapy. Nat. Rev. Cancer.

[26] Pawelczyk K., Piotrowska A., Ciesielska U., Jablonska K., Gletzel-Plucinska N., Grzegrzolka J., Podhorska-Okolow M., Dziegiel P., Nowinska K. (2019). Role of PD-L1 expression in non-small cell lung cancer and their prognostic significance according to clinicopathological factors and diagnostic markers. Int. J. Mol. Sci..

[27] Vaddepally R.K., Kharel P., Pandey R., Garje R., Chandra A.B. (2020). Review of indications of FDA—Approved immune checkpoint inhibitors per NCCN guidelines with the level of evidence. Cancers.

[28] Melosky B., Cheema P.K., Brade A., McLeod D., Liu G., Price P.W., Jao K., Schellenberg D.D., Juergens R., Leighl N. (2020). Prolonging survival: The role of immune checkpoint inhibitors in the treatment of extensive-stage small cell lung cancer. Oncologist.

[29] Garon E.B., Hellmann M.D., Rizvi N.A., Carcereny E., Leighl N.B., Ahn M.J., Eder J.P., Balmanoukian A.S., Aggarwal C., Horn L. (2019). Five-year overall survival for patients with advanced non‒small-cell lung cancer treated with pembrolizumab: Results from the phase I KEYNOTE-001 study. J. Clin. Oncol..

[30] Brueckl W.M., Ficker J.H., Zeitler G. (2020). Clinically relevant prognostic and predictive markers for immune-checkpoint-inhibitor (ICI) therapy in non-small cell lung cancer (NSCLC). BMC Cancer.

[31] Siegel R.L., Miller K.D., Jemal A. (2017). Cancer statistics, 2017. CA Cancer J. Clin..

[32] Fulda S., Debatin K.M. (2006). Extrinsic versus intrinsic apoptosis pathways in anticancer chemotherapy. Oncogene.

[33] Rosato A., Menin C., Boldrin D., Dalla Santa S., Bonaldi L., Scaini M.C., Del Bianco P., Zardo D., Fassan M., Cappellesso R. (2013). Survivin expression impacts prognostically on NSCLC but not SCLC. Lung Cancer.

[34] Ikeguchi M., Ueda T., Sakatani T., Hirooka Y., Kaibara N. (2002). Expression of survivin messenger RNA correlates with poor prognosis in patients with hepatocellular carcinoma. Diagn. Mol. Pathol..

[35] Lu C.D., Altieri D.C., Tanigawa N. (1998). Expression of a novel antiapoptosis gene, survivin, correlated with tumor cell apoptosis and p53 accumulation in gastric carcinomas. Cancer Res..

[36] Sarela A.I., Macadam R.C., Farmery S.M., Markham A.F., Guillou P.J. (2000). Expression of the antiapoptosis gene, survivin, predicts death from recurrent colorectal carcinoma. Gut.

[37] Chen P., Li J., Ge L.P., Dai C.H., Li X.Q. (2010). Prognostic value of survivin, X-linked inhibitor of apoptosis protein and second mitochondria-derived activator of caspases expression in advanced non-small-cell lung cancer patients. Respirology.

[38] Rathore R., McCallum J.E., Varghese E., Florea A.M., Büsselberg D. (2017). Overcoming chemotherapy drug resistance by targeting inhibitors of apoptosis proteins (IAPs). Apoptosis.

[39] Huang Y., Park Y.C., Rich R.L., Segal D., Myszka D.G., Wu H. (2001). Structural basis of caspase inhibition by XIAP: Differential roles of the linker versus the BIR domain. Cell.

[40] Garg H., Suri P., Gupta J.C., Talwar G.P., Dubey S. (2016). Survivin: A unique target for tumor therapy. Cancer Cell Int..

[41] Shinohara E.T., Gonzalez A., Massion P.P., Chen H., Li M., Freyer A.S., Olson S.J., Andersen J.J., Shyr Y., Carbone D.P. (2005). Nuclear survivin predicts recurrence and poor survival in patients with resected nonsmall cell lung carcinoma. Cancer.

[42] Bria E., Visca P., Novelli F., Casini B., Diodoro M.G., Perrone-Donnorso R., Botti C., Sperduti I., Facciolo F., Milella M. (2008). Nuclear and cytoplasmic cellular distribution of survivin as survival predictor in resected non-small-cell lung cancer. Eur. J. Surg. Oncol..

[43] Hu S., Qu Y., Xu X., Xu Q., Geng J., Xu J. (2013). Nuclear survivin and its relationship to DNA damage repair genes in non-small cell lung cancer investigated using tissue array. PLoS ONE.

[44] Li F., Ackermann E.J., Bennett C.F., Rothermel A.L., Plescia J., Tognin S., Villa A., Marchisio P.C., Altieri D.C. (1999). Pleiotropic cell-division defects and apoptosis induced by interference with survivin function. Nat. Cell Biol..

[45] Dohi T., Okada K., Xia F., Wilford C.E., Samuel T., Welsh K., Marusawa H., Zou H., Armstrong R., Matsuzawa S. (2004). An IAP-IAP complex inhibits apoptosis. J. Biol. Chem..

[46] Mehrotra S., Languino L.R., Raskett C.M., Mercurio A.M., Dohi T., Altieri D.C. (2010). IAP regulation of metastasis. Cancer Cell.

[47] Raj D., Liu T., Samadashwily G., Li F., Grossman D. (2008). Survivin repression by p53, Rb and E2F2 in normal human melanocytes. Carcinogenesis.

[48] Seo S.K., Hwang C.S., Choe T.B., Hong S.I., Yi J.Y., Hwang S.G., Lee H.G., Oh S.T., Lee Y.H., Park I.C. (2015). Selective inhibition of histone deacetylase 2 induces p53-dependent survivin downregulation through MDM2 proteasomal degradation. Oncotarget.

[49] Tran J., Master Z., Yu J.L., Rak J., Dumont D.J., Kerbel R.S. (2002). A role for survivin in chemoresistance of endothelial cells mediated by VEGF. Proc. Natl. Acad. Sci. USA.

[50] Akyürek N., Memiş L., Ekinci O., Köktürk N., Oztürk C. (2006). Survivin expression in pre-invasive lesions and non-small cell lung carcinoma. Virchows Arch..

[51] Karczmarek-Borowska B., Filip A., Wojcierowski J., Smoleń A., Pilecka I., Jabłonka A. (2005). Survivin antiapoptotic gene expression as a prognostic factor in non-small cell lung cancer: In situ hybridization study. Folia Histochem. Cytobiol..

[52] Dai C.H., Li J., Shi S.B., Yu L.C., Ge L.P., Chen P. (2010). Survivin and smac gene expressions but not livin are predictors of prognosis in non-small cell lung cancer patients treated with adjuvant chemotherapy following surgery. Jpn. J. Clin. Oncol..

[53] Dong X., Lin D., Low C., Vucic E.A., English J.C., Yee J., Murray N., Lam W.L., Ling V., Lam S. (2013). Elevated expression of BIRC6 protein in non-small-cell lung cancers is associated with cancer recurrence and chemoresistance. J. Thorac. Oncol..

[54] Kren L., Brazdil J., Hermanova M., Goncharuk V.N., Kallakury B.V., Kaur P., Ross J.S. (2004). Prognostic significance of anti-apoptosis proteins survivin and bcl-2 in non-small cell lung carcinomas: A clinicopathologic study of 102 cases. Appl. Immunohistochem. Mol. Morphol..

[55] Liang Y., Wang H., Sun Y., Chen S., Wang H., Huang R., Zhao X., Fu W., Yang C. (2017). miR-198-induced upregulation of Livin may be associated with the prognosis and contribute to the oncogenesis of lung adenocarcinoma. Oncol. Rep..

[56] Richardson W.S., Wilson M.C., Nishikawa J., Hayward R.S. (1995). The well-built clinical question: A key to evidence-based decisions. ACP J. Club.

[57] Shea B.J., Grimshaw J.M., Wells G.A., Boers M., Andersson N., Hamel C., Porter A.C., Tugwell P., Moher D., Bouter L.M. (2007). Development of AMSTAR: A measurement tool to assess the methodological quality of systematic reviews. BMC Med. Res. Methodol..

[58] Moher D., Liberati A., Tetzlaff J., Altman D.G. (2009). Preferred reporting items for systematic reviews and meta-analyses: The PRISMA statement. PLoS Med..

[59] Steels E., Paesmans M., Berghmans T., Branle F., Lemaitre F., Mascaux C., Meert A.P., Vallot F., Lafitte J.J., Sculier J.P. (2001). Role of p53 as a prognostic factor for survival in lung cancer: A systematic review of the literature with a meta-analysis. Eur. Respir. J..

[60] Nagy Á., Munkácsy G., Győrffy B. (2021). Pancancer survival analysis of cancer hallmark genes. Sci. Rep..

[61] Lau J., Ioannidis J.P., Schmid C.H. (1997). Quantitative synthesis in systematic reviews. Ann. Intern. Med..

[62] Higgins J.P., Thompson S.G. (2002). Quantifying heterogeneity in a meta-analysis. Stat. Med..

[63] DerSimonian R., Laird N. (1986). Meta-analysis in clinical trials. Control. Clin. Trials.

[64] Bartoš F., Maier M., Wagenmakers E.-J. (2020). Adjusting for publication bias in JASP—Selection models and robust bayesian meta-analysis. PsyArXiv.

[65] Yano Y., Otsuka T., Hirano H., Uenami T., Satomi A., Kuroyama M., Niinaka M., Yoneda T., Kimura H., Mori M. (2015). Nuclear survivin expression in small cell lung cancer. Anticancer Res..

[66] Chen P., Zhu J., Liu D.Y., Li H.Y., Xu N., Hou M. (2014). Over-expression of survivin and VEGF in small-cell lung cancer may predict the poorer prognosis. Med. Oncol..

[67] Yoo J., Jung J.H., Lee M.A., Seo K.J., Shim B.Y., Kim S.H., Cho D.G., Ahn M.I., Kim C.H., Cho K.D. (2007). Immunohistochemical analysis of non-small cell lung cancer: Correlation with clinical parameters and prognosis. J. Korean Med. Sci..

[68] Monzó M., Rosell R., Felip E., Astudillo J., Sánchez J.J., Maestre J., Martín C., Font A., Barnadas A., Abad A. (1999). A novel anti-apoptosis gene: Re-expression of survivin messenger RNA as a prognosis marker in non-small-cell lung cancers. J. Clin. Oncol..

[69] Sun P.L., Jin Y., Kim H., Seo A.N., Jheon S., Lee C.T., Chung J.H. (2013). Survivin expression is an independent poor prognostic marker in lung adenocarcinoma but not in squamous cell carcinoma. Virchows Arch..

[70] Xu P., Xu X.L., Huang Q., Zhang Z.H., Zhang Y.B. (2012). CIP2A with survivin protein expressions in human non-small-cell lung cancer correlates with prognosis. Med. Oncol..

[71] Gao Q., Yang S., Kang M.Q. (2012). Influence of survivin and Bcl-2 expression on the biological behavior of non-small cell lung cancer. Mol. Med. Rep..

[72] Mohamed S., Yasufuku K., Nakajima T., Hiroshima K., Chiyo M., Yoshida S., Suzuki M., Sekine Y., Shibuya K., Agamy G. (2009). Nuclear survivin in pN2 nonsmall cell lung cancer: Prognostic and clinical implications. Eur. Respir. J..

[73] Cho S., Park T.I., Lee E.B., Son S.A. (2012). Poor prognostic factors in surgically resected stage I non-small cell lung cancer: Histopathologic and immunohistochemical analysis. Korean J. Thorac. Cardiovasc. Surg..

[74] Vischioni B., van der Valk P., Span S.W., Kruyt F.A., Rodriguez J.A., Giaccone G. (2004). Nuclear localization of survivin is a positive prognostic factor for survival in advanced non-small-cell lung cancer. Ann. Oncol..

[75] Wang M., Liu B.G., Yang Z.Y., Hong X., Chen G.Y. (2012). Significance of survivin expression: Prognostic value and survival in stage III non-small cell lung cancer. Exp. Ther. Med..

[76] Wu Y.K., Huang C.Y., Yang M.C., Lan C.C., Lee C.H., Chan E.C., Chen K.T. (2014). Nuclear survivin expression: A prognostic factor for the response to taxane-platinum chemotherapy in patients with advanced non-small cell lung cancer. Med. Oncol..

[77] Yamashita S., Chujo M., Miyawaki M., Tokuishi K., Anami K., Yamamoto S., Kawahara K. (2009). Combination of p53AIP1 and survivin expression is a powerful prognostic marker in non-small cell lung cancer. J. Exp. Clin. Cancer Res..

[78] Cho H.J., Kim H.R., Park Y.S., Kim Y.H., Kim D.K., Park S.I. (2015). Prognostic value of survivin expression in stage III non-small cell lung cancer patients treated with platinum-based therapy. Surg. Oncol..

[79] Falleni M., Pellegrini C., Marchetti A., Oprandi B., Buttitta F., Barassi F., Santambrogio L., Coggi G., Bosari S. (2003). Survivin gene expression in early-stage non-small cell lung cancer. J. Pathol..

[80] Hirano H., Maeda H., Takeuchi Y., Susaki Y., Kobayashi R., Hayashi A., Ose N., Nakazawa Y., Yamaguchi T., Yokota S. (2014). Association of cigarette smoking with the expression of nuclear survivin in pathological Stage IA lung adenocarcinomas. Med. Mol. Morphol..

[81] Porebska I., Sobańska E., Kosacka M., Jankowska R. (2010). Apoptotic regulators: P53 and survivin expression in non-small cell lung cancer. Cancer Genom. Proteom..

[82] Nakashima N., Huang C.L., Liu D., Ueno M., Yokomise H. (2010). Intratumoral Wnt1 expression affects survivin gene expression in non-small cell lung cancer. Int. J. Oncol..

[83] Atikcan S., Unsal E., Demirag F., Köksal D., Yilmaz A. (2006). Correlation between survivin expression and prognosis in non-small cell lung cancer. Respir. Med..

[84] Huang W., Mao Y., Zhan Y., Huang J., Wang X., Luo P., Li L.I., Mo D., Liu Q., Xu H. (2016). Prognostic implications of survivin and lung resistance protein in advanced non-small cell lung cancer treated with platinum-based chemotherapy. Oncol. Lett..

[85] Kim G.Y., Lim S.J., Kim Y.W. (2011). Expression of HuR, COX-2, and survivin in lung cancers; cytoplasmic HuR stabilizes cyclooxygenase-2 in squamous cell carcinomas. Mod. Pathol..

[86] Yang D.X., Li N.E., Ma Y., Han Y.C., Shi Y. (2010). Expression of Elf-1 and survivin in non-small cell lung cancer and their relationship to intratumoral microvessel density. Chin. J. Cancer.

[87] Chen Y.Q., Zhao C.L., Li W. (2009). Effect of hypoxia-inducible factor-1alpha on transcription of survivin in non-small cell lung cancer. J. Exp. Clin. Cancer Res..

[88] Ikehara M., Oshita F., Kameda Y., Ito H., Ohgane N., Suzuki R., Saito H., Yamada K., Noda K., Mitsuda A. (2002). Expression of survivin correlated with vessel invasion is a marker of poor prognosis in small adenocarcinoma of the lung. Oncol. Rep..

[89] Fan C.F., Xu H.T., Lin X.Y., Yu J.H., Wang E.H. (2011). A multiple marker analysis of apoptosis-associated protein expression in non-small cell lung cancer in a Chinese population. Folia Histochem. Cytobiol..

[90] Yu S., Zhang Z., Zhang B., Shu Y., Wu H., Huang X., Yu Q., Guo R. (2014). Clinical significance of PIK3CA and survivin in primary adenosquamous lung carcinoma. Med. Oncol..

[91] Li C., Wang L., Zheng L., Zhan X., Xu B., Jiang J., Wu C. (2015). SIRT1 expression is associated with poor prognosis of lung adenocarcinoma. Onco Targets Ther..

[92] Grossi F., Spizzo R., Bordo D., Cacitti V., Valent F., Rossetto C., Follador A., Di Terlizzi S., Aita M., Morelli A. (2010). Prognostic stratification of stage IIIA pN2 non-small cell lung cancer by hierarchical clustering analysis of tissue microarray immunostaining data: An alpe adria thoracic oncology multidisciplinary group study (ATOM 014). J. Thorac. Oncol..

[93] Wang X.Y., Yao Z., Li Y., Liu T., Zheng H.Y., Zhu C.Z., Sun C.Y., Wang A.X., Zhao M., Wu X.Y. (2005). Expression and significance of survivin mRNA in lung cancer tissue microarray detected by FISH. Chin. Med. Sci. J..

[94] Xia R., Chen S., Chen Y., Zhang W., Zhu R., Deng A. (2015). A chromosomal passenger complex protein signature model predicts poor prognosis for non-small-cell lung cancer. OncoTargets Ther..

[95] Ferreira C.G., van der Valk P., Span S.W., Ludwig I., Smit E.F., Kruyt F.A., Pinedo H.M., van Tinteren H., Giaccone G. (2001). Expression of X-linked inhibitor of apoptosis as a novel prognostic marker in radically resected non-small cell lung cancer patients. Clin. Cancer Res..

[96] Hofmann H.S., Simm A., Hammer A., Silber R.E., Bartling B. (2002). Expression of inhibitors of apoptosis (IAP) proteins in non-small cell human lung cancer. J. Cancer Res. Clin. Oncol..

[97] Gharabaghi M.A. (2018). Diagnostic investigation of BIRC6 and SIRT1 protein expression level as potential prognostic biomarkers in patients with non-small cell lung cancer. Clin. Respir. J..

[98] Sun L., Wang Y., Yuan H., Burnett J., Pan J., Yang Z., Ran Y., Myers I., Sun D. (2016). CPA4 is a novel diagnostic and prognostic marker for human non-small-cell lung cancer. J. Cancer.

[99] Liu J.L., Zhang X.J., Zhang Z., Zhang A.H., Wang W., Dong J.H. (2013). Meta-analysis: Prognostic value of survivin in patients with hepatocellular carcinoma. PLoS ONE.

[100] Fan J., Wang L., Jiang G.N., He W.X., Ding J.A. (2008). The role of survivin on overall survival of non-small cell lung cancer, a meta-analysis of published literatures. Lung Cancer.

[101] Song Z., Yao X., Wu M. (2003). Direct interaction between survivin and Smac/DIABLO is essential for the anti-apoptotic activity of survivin during taxol-induced apoptosis. J. Biol. Chem..

[102] Du C., Fang M., Li Y., Li L., Wang X. (2000). Smac, a mitochondrial protein that promotes cytochrome c-dependent caspase activation by eliminating IAP inhibition. Cell.

[103] Chai J., Du C., Wu J.W., Kyin S., Wang X., Shi Y. (2000). Structural and biochemical basis of apoptotic activation by Smac/DIABLO. Nature.

[104] Yang C., Wang H., Zhang B., Chen Y., Zhang Y., Sun X., Xiao G., Nan K., Ren H., Qin S. (2016). LCL161 increases paclitaxel-induced apoptosis by degrading cIAP1 and cIAP2 in NSCLC. J. Exp. Clin. Cancer Res..

[105] Tolcher A.W., Mita A., Lewis L.D., Garrett C.R., Till E., Daud A.I., Patnaik A., Papadopoulos K., Takimoto C., Bartels P. (2008). Phase I and pharmacokinetic study of YM155, a small-molecule inhibitor of survivin. J. Clin. Oncol..

[106] Rauch A., Hennig D., Schäfer C., Wirth M., Marx C., Heinzel T., Schneider G., Krämer O.H. (2014). Survivin and YM155: How faithful is the liaison?. Biochim. Biophys. Acta.

[107] Giaccone G., Zatloukal P., Roubec J., Floor K., Musil J., Kuta M., van Klaveren R.J., Chaudhary S., Gunther A., Shamsili S. (2009). Multicenter phase II trial of YM155, a small-molecule suppressor of survivin, in patients with advanced, refractory, non-small-cell lung cancer. J. Clin. Oncol..

[108] Kelly R.J., Thomas A., Rajan A., Chun G., Lopez-Chavez A., Szabo E., Spencer S., Carter C.A., Guha U., Khozin S. (2013). A phase I/II study of sepantronium bromide (YM155, survivin suppressor) with paclitaxel and carboplatin in patients with advanced non-small-cell lung cancer. Ann. Oncol..

[109] Shimizu T., Nishio K., Sakai K., Okamoto I., Okamoto K., Takeda M., Morishita M., Nakagawa K. (2020). Phase I safety and pharmacokinetic study of YM155, a potent selective survivin inhibitor, in combination with erlotinib in patients with EGFR TKI refractory advanced non-small cell lung cancer. Cancer Chemother. Pharmacol..

[110] Sun Y., Giacalone N.J., Lu B. (2011). Terameprocol (tetra-O-methyl nordihydroguaiaretic acid), an inhibitor of Sp1-mediated survivin transcription, induces radiosensitization in non-small cell lung carcinoma. J. Thorac. Oncol..

[111] Rödel F., Frey B., Leitmann W., Capalbo G., Weiss C., Rödel C. (2008). Survivin antisense oligonucleotides effectively radiosensitize colorectal cancer cells in both tissue culture and murine xenograft models. Int. J. Radiat. Oncol. Biol. Phys..

[112] Hansen J.B., Fisker N., Westergaard M., Kjaerulff L.S., Hansen H.F., Thrue C.A., Rosenbohm C., Wissenbach M., Orum H., Koch T. (2008). SPC3042: A proapoptotic survivin inhibitor. Mol. Cancer Ther..

[113] Sebastian M., Schröder A., Scheel B., Hong H.S., Muth A., von Boehmer L., Zippelius A., Mayer F., Reck M., Atanackovic D. (2019). A phase I/IIa study of the mRNA-based cancer immunotherapy CV9201 in patients with stage IIIB/IV non-small cell lung cancer. Cancer Immunol. Immunother. CII.

[114] Ge C., Li R., Song H., Geng T., Yang J., Tan Q., Song L., Wang Y., Xue Y., Li Z. (2017). Phase I clinical trial of a novel autologous modified-DC vaccine in patients with resected NSCLC. BMC Cancer.

