# Supplementary material for: Clinicopathological and Prognostic Significance of Inhibitor of Apoptosis Protein (IAP) Family Members in Lung Cancer: A Meta-Analysis
Source: Cancers (Basel). 2021 Aug 14;13(16):4098. doi: 10.3390/cancers13164098 (PMC8392569; doi:10.3390/cancers13164098)
Supplement: Supplementary file 1 [file cancers-13-04098-s001.zip › Supplementary Figures.pdf]

Study or Subgroup	log[Hazard Ratio]	SE	Weight	Hazard Ratio IV, Random, 95% CI	Year
**9.1.1 Cytoplasmic**					
Kren L	1.1378	0.2372	3.8%	3.12 [1.96, 4.97]	2004
Shinohara T (Cyt)	-0.2231	0.4366	2.6%	0.80 [0.34, 1.88]	2005
Akyürek N	1.0852	0.2951	3.5%	2.96 [1.66, 5.28]	2006
Bria E (Cyt)	0.0862	0.256	3.7%	1.09 [0.66, 1.80]	2008
Porebska I	0.131	0.3448	3.2%	1.14 [0.58, 2.24]	2010
Grossi F	0.4762	0.2746	3.6%	1.61 [0.94, 2.76]	2010
Kim GY (ADC-Cyt)	1.5063	0.4948	2.4%	4.51 [1.71, 11.89]	2011
Kim GY (SCC-Cyt)	0.7178	0.2949	3.5%	2.05 [1.15, 3.65]	2011
Fan CF	0.8372	0.2741	3.6%	2.31 [1.35, 3.95]	2011
Wang M	0.6678	0.1876	4.1%	1.95 [1.35, 2.82]	2012
Sun PL (SCC)	0.3001	0.2606	3.7%	1.35 [0.81, 2.25]	2013
Sun PL (ADC)	0.678	0.2927	3.5%	1.97 [1.11, 3.50]	2013
Chen P (2)	1.5141	0.5843	2.0%	4.55 [1.45, 14.29]	2014
Huang W	0.5766	0.2791	3.5%	1.78 [1.03, 3.08]	2016
**Subtotal (95% CI)**			**46.4%**	**1.90 [1.52, 2.36]**	
Heterogeneity: Tau² = 0.08; Chi² = 25.48, df = 13 (P = 0.02); I² = 49%					
Test for overall effect: Z = 5.75 (P < 0.00001)					
**9.1.2 Nuclear**					
Shinohara T (Nuc)	1.008	0.3844	2.9%	2.74 [1.29, 5.82]	2005
Bria E (Nuc)	0.6043	0.3033	3.4%	1.83 [1.01, 3.32]	2008
Hu S	0.5188	0.2596	3.7%	1.68 [1.01, 2.79]	2013
Xia R	0.7227	0.339	3.2%	2.06 [1.06, 4.00]	2015
Yano Y	-0.0408	0.0273	4.6%	0.96 [0.91, 1.01]	2015
**Subtotal (95% CI)**			**17.8%**	**1.64 [1.03, 2.63]**	
Heterogeneity: Tau² = 0.21; Chi² = 21.08, df = 4 (P = 0.0003); I² = 81%					
Test for overall effect: Z = 2.08 (P = 0.04)					
**9.1.3 Cytoplasmic and nuclear**					
Vischioni B	-0.7985	0.3651	3.0%	0.45 [0.22, 0.92]	2004
Atikcan S	1.3164	0.4547	2.6%	3.73 [1.53, 9.09]	2006
Mohamed S	0.793	0.2886	3.5%	2.21 [1.26, 3.89]	2009
Nakashima N	0.6313	0.3221	3.3%	1.88 [1.00, 3.53]	2010
Gao Q	1.0438	0.3156	3.3%	2.84 [1.53, 5.27]	2012
Wu YK	1.8181	0.7416	1.5%	6.16 [1.44, 26.35]	2014
**Subtotal (95% CI)**			**17.2%**	**2.05 [1.08, 3.88]**	
Heterogeneity: Tau² = 0.47; Chi² = 22.18, df = 5 (P = 0.0005); I² = 77%					
Test for overall effect: Z = 2.21 (P = 0.03)					
**9.1.4 No information**					
Ikehara M	1.4861	0.4555	2.5%	4.42 [1.81, 10.79]	2002
Falleni M	0.0862	0.3976	2.9%	1.09 [0.50, 2.38]	2003
Xu P	0.8286	0.2474	3.7%	2.29 [1.41, 3.72]	2012
Cho HJ	0.9282	0.4536	2.6%	2.53 [1.04, 6.15]	2015
Li C	0.2744	0.3673	3.0%	1.32 [0.64, 2.70]	2015
Sun L	-0.1508	0.2318	3.8%	0.86 [0.55, 1.35]	2016
**Subtotal (95% CI)**			**18.6%**	**1.69 [1.03, 2.77]**	
Heterogeneity: Tau² = 0.25; Chi² = 16.38, df = 5 (P = 0.006); I² = 69%					
Test for overall effect: Z = 2.10 (P = 0.04)					
**Total (95% CI)**			**100.0%**	**1.84 [1.49, 2.27]**	
Heterogeneity: Tau² = 0.25; Chi² = 173.92, df = 30 (P < 0.00001); I² = 83%					
Test for overall effect: Z = 5.62 (P < 0.00001)					
Test for subgroup differences: Chi² = 0.51, df = 3 (P = 0.92), I² = 0%					

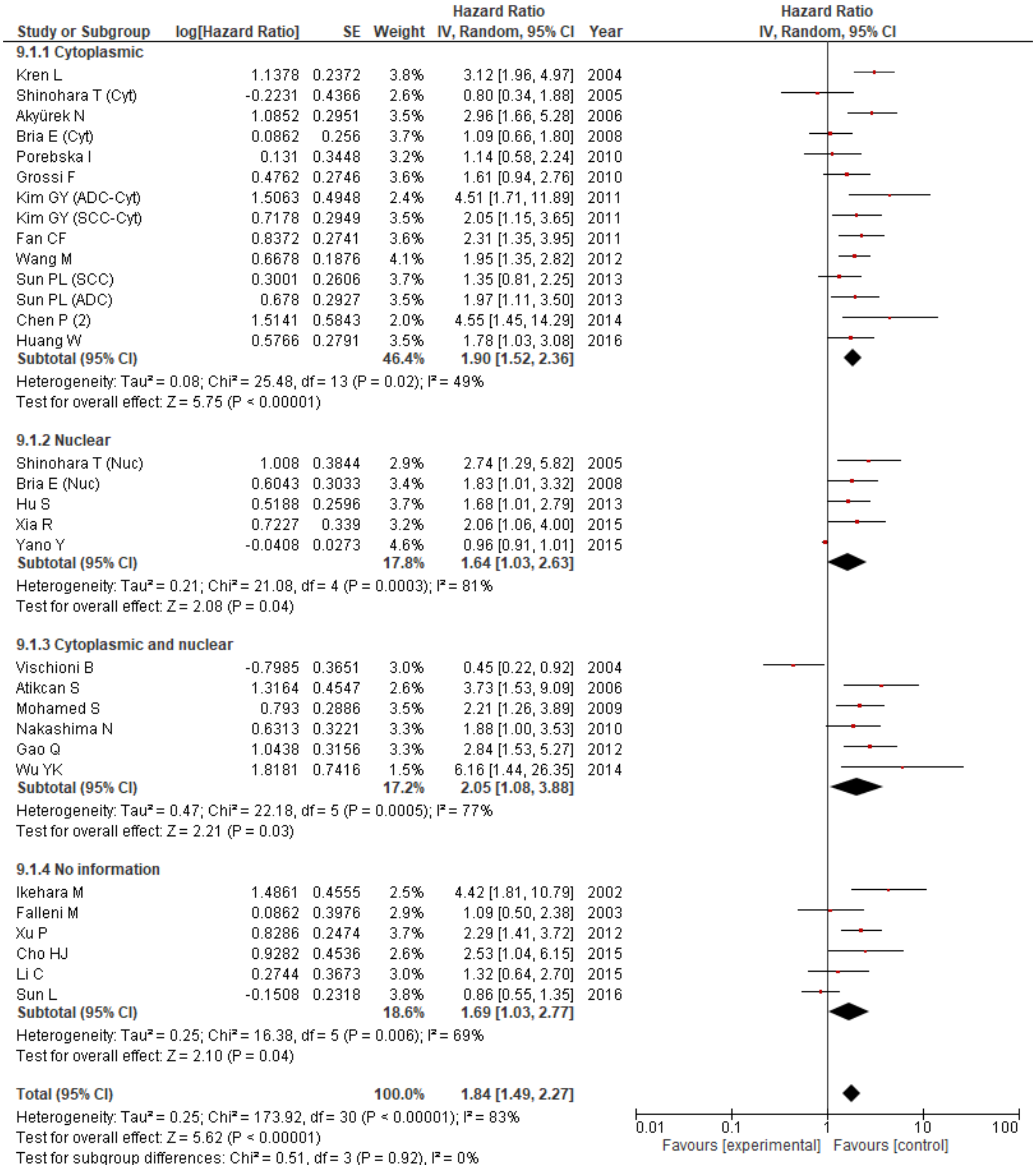

Figure S1. Meta-analysis comparing the subcellular expression of survivin with OS in patients with lung cancer. Forest plot reflects the individual and pooled HR with 95% CI. Heterogeneity was quantified by the Cochrane Q test (Chi-squared test; Chi²) and inconsistency (I²).

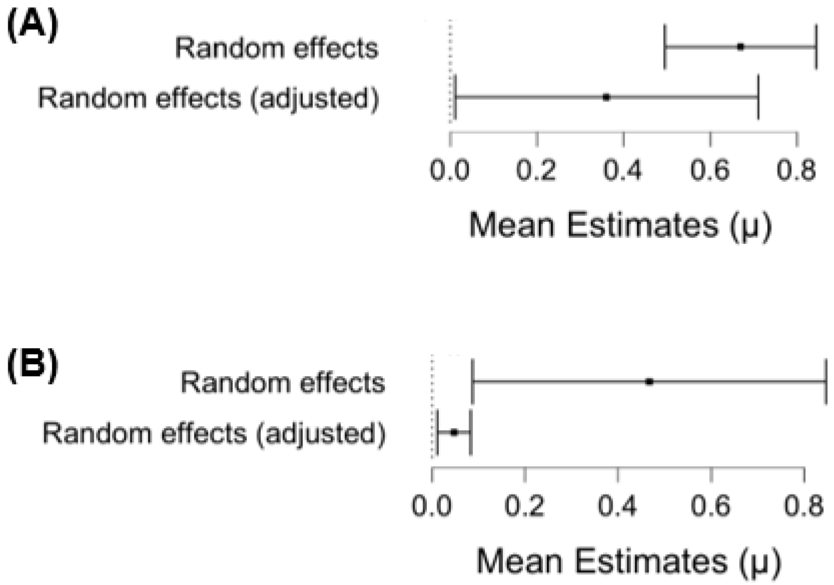

Figure S2. Comparison of effect size estimates of the adjusted/unadjusted random effects model for the association of survivin expression and (A) OS or (B) DFS. After adjusting for publication bias the estimated effect sizes are still statistically significant. Mean estimates (μ) corresponds to the log [Hazard Ratio].

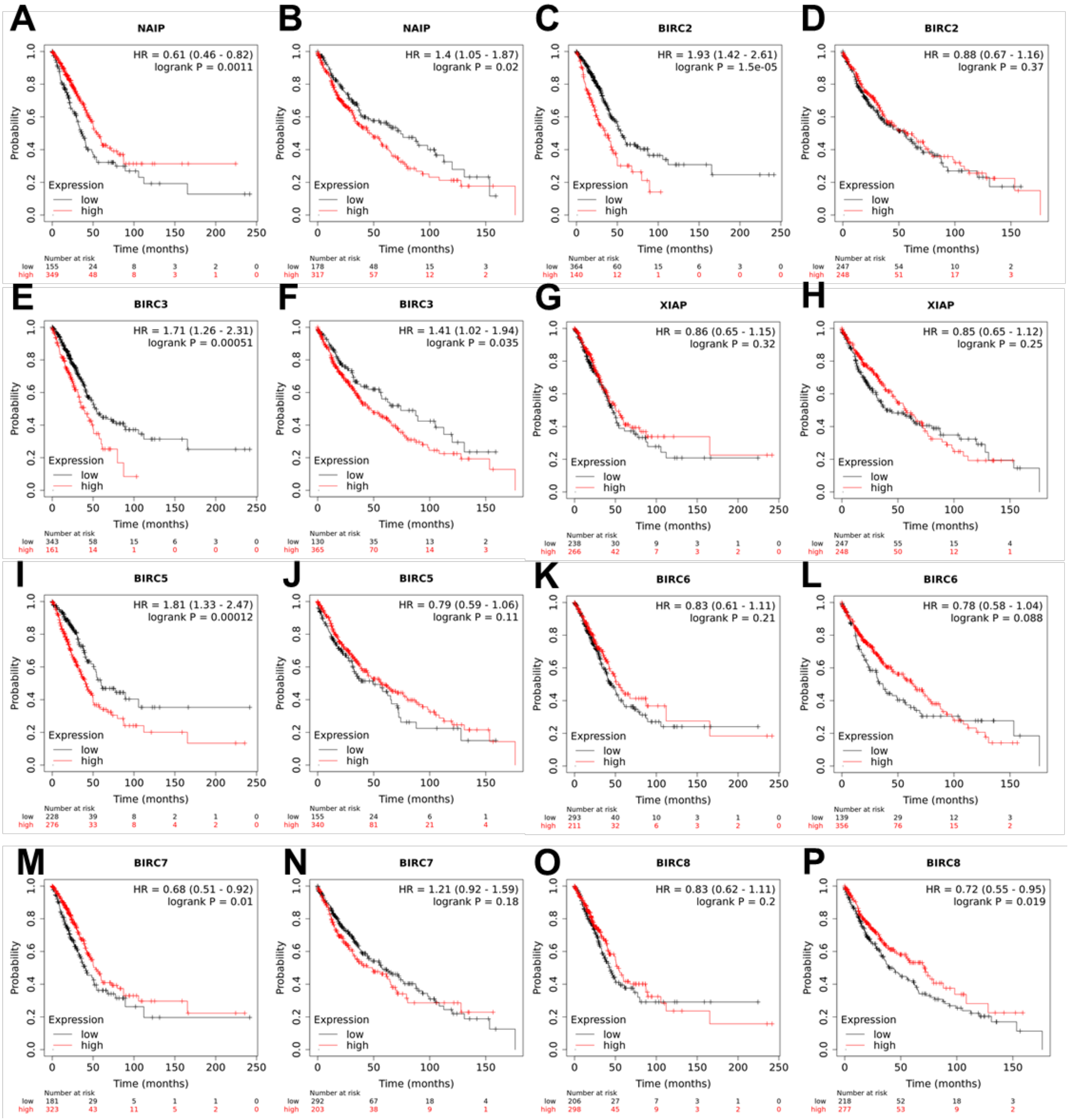

Figure S3. Kaplan-Meier curves for overall survival (OS) in lung cancer patients depending on the IAP/BIRC family member. The TCGA dataset was used to analyse the expression of **(A, B)** NAIP/BIRC1, **(C, D)** cIAP1/BIRC2, **(E, F)** cIAP2/BIRC3, **(G, H)** XIAP/BIRC4, **(I, J)** survivin/BIRC5, **(K, L)** BRUCE/BIRC6, **(M, N)** livin/BIRC7 and **(O, P)** Ts-IAP/BIRC8 in **(A, C, E, G, I, K, M, O)** lung adenocarcinoma (LUAD) or **(B, D, F, H, J, L, N, P)** lung squamous cell carcinoma (LUSC).

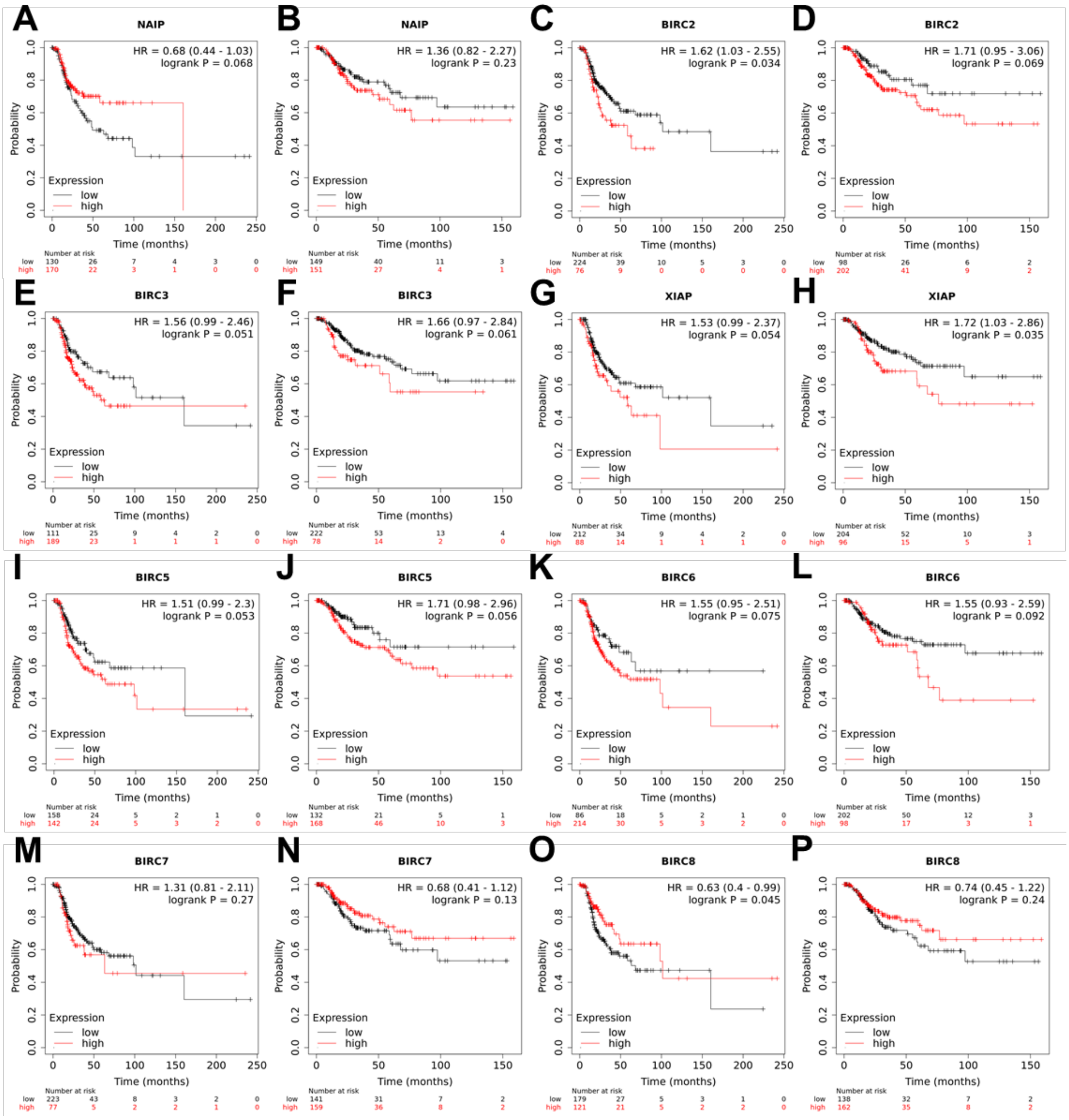

Figure S4. Kaplan-Meier curves for disease free survival (DFS) in lung cancer patients depending on the IAP/BIRC family member. The TCGA dataset was used to analyse the expression of **(A, B)** NAIP/BIRC1, **(C, D)** cIAP1/BIRC2, **(E, F)** cIAP2/BIRC3, **(G, H)** XIAP/BIRC4, **(I, J)** survivin/BIRC5, **(K, L)** BRUCE/BIRC6, **(M, N)** livin/BIRC7 and **(O, P)** Ts-IAP/BIRC8 in **(A, C, E, G, I, K, M, O)** lung adenocarcinoma (LUAD) or **(B, D, F, H, J, L, N, P)** lung squamous cell carcinoma (LUSC).
