# Supplementary material for: Clinicopathological and Prognostic Significance of Inhibitor of Apoptosis Protein (IAP) Family Members in Lung Cancer: A Meta-Analysis
Source: Cancers (Basel). 2021 Aug 14;13(16):4098. doi: 10.3390/cancers13164098 (PMC8392569; doi:10.3390/cancers13164098)
Supplement: Supplementary file 1 [file cancers-13-04098-s001.zip › Supplementary Tables.pdf]

Table S1. Methodological characteristics of included studies.

First Author	Marker	Sample storage	Method	Antibody (Dilution)	Cut off value	Expression (%)	Subcellular localization	Analysed Variables	Outcome	HR Estimate	HR	95% CI
Yano Y. [66]	SVV	NA	IHC	Rabbit pAb, Novus Biologicals (1:500)	>10% of LI	96.8	Nuc	(A, S, BI, TS, U)[NE]	OS	MV	0.96	0.91-1.02
Karczmarek - Borowska B. [51]	SVV	FFPE	FISH	NA	NA	58.3		S, PS, U, HT, D, RC, nCT	OS	MV	4.27	0.39-1.45
Rosato A. [33]												
(NSCLC)	SVV	FFPE	RT-PCR	NA	>3.96 fold	NA		(S, HT, D, U, N, SNP)[NE]	OS	MV	5.5	1.76-17.15
(SCLC)	SVV	FFPE	RT-PCR	NA	>3.96 fold	NA		(S, IASCLC, SNP)[NE]	NA	NA	NA	NA
Chen P. (1) [37]	SVV<br>XIAP	FFT	RT-PCR	NA	Median expression level	NA		(A, S, PS, WL, HT, G, U, CR)[NE]	OS+PFS	MV	OS: 2.28 PFS: 2.13<br>OS: 0.63 PFS: 0.79	OS:0.96-3.31<br>PFS:0.82-3.26<br>OS: 0.31-1.17<br>PFS:0.40-1.12
Dai C.H. [52]	SVV<br>Livinα:<br>Livinβ:	FFT	RT-PCR	NA	Median expression level	50		(A, S, Smo, HT, G, U, N)[NE]	OS+TFS	MV<br>UV (SC)<br>UV (SC)	OS: 2.38 TFS: 2.26<br>OS:0.89<br>OS: 0.88	OS:0.91-3.54<br>TFS:0.85-3.28<br>OS: 0.52-1.48<br>OS:0.52-1.46
Bria E. [42]	SVV	FFPE	IHC	pAb, Ab469, Abcam	≥20%	Nuc: 72.4 Cyt: 71.6	Cyt + Nuc	A, S, HT, Rt, T, N	OS	MV<br>UV	Nuc: 1.83<br>Cyt: 1.09	Nuc: 1.01-3.30<br>Cyt: 0.66-1.78
Yoo J. [68]	SVV	FFPE	IHC	clone 4F7, NeoMarkers 1:50	>10%	2.7	NA	HT, G, T, N, U	DSS	MV	0.98	0.22-4.29
Monzó M. [69]	SVV	FFT	RT-PCR	NA	NA	85.5		A, S, T, N, U, HT	OS	UV	2.20	1.10-4.50

Sun P.L. [70]	SVV	FFPE	IHC	Santa Cruz Biotechnology	Median Score (staining	ADC:52.9<br>SCC:54.1	Cyt > Cyt+Nuc	A, S, Smo, TS, Pi, Vi, Li, Pn, N, U, Rec, AT	OS+DFS	ADC: MV<br>SCC:	ADC: OS: 1.97<br>DFS: 1.69	ADC: OS:<br>1.11-3.49 DFS:<br>1.12-2.53

					intensity + percentage)					UV (SC)	SCC: OS: 1.35 DFS: 0.92	SCC: OS: 0.81 – 2.27 DFS: 0.59-1.45
Xu P. [71]	SVV	FFPE	IHC	Rabbit pAb, Bioworld, 1:100	H-score ≥ 1 staining intensity + percentage)	66	NA	A, S, HT, D, U, T, N	OS	UV (SC)	2.29	1.41-3.72
Gao Q. [72]	SVV	FFPE	IHC	Rabbit pAb, Fuzhou Maixin Biotechnology	H-score > 1 (staining intensity + percentage)	58.1	Cyt + Nuc	A, S, HT, TS, G, N, U	OS	UV (SC)	2.84	1.53-5.28
Chen P. (2) [67]	SVV	FFPE	IHC	NA	Score ≥ 2 (staining intensity + percentage)	73.3	Cyt	A, S, TS, N, Smo, TT, U	OS	MV	0.22 (neg. vs. pos.)	0.07- 0,68
Mohamed S. [73]	SVV	FFPE	IHC	Mouse mAb, DakoCytomation, 1:400	>10%	87.2 Nuc: 60.3	Nuc + Cyt	NA	OS	MV	2.21	0.26-0.80
Hu S. [43]	SVV	FFPE	IHC	Rabbit mAb 71G4B7, Cell Signaling, 1:400	Median Score (staining intensity + percentage)	51.6	Nuc	A, S, HT, G, U, T, N	OS+DFS	MV	OS: 1.68 DFS: 1.83	OS: 1.01- 2.81 DFS: 1.03-3.26
Shinohara E.T. [41]	SVV	FFPE	IHC	mAb, sc-17779, Santa Cruz Biotechnology, 1:50	Weak 1+ (staining intensity)	Nuc: 74.5 Cyt: 17.7	Nuc + Cyt	A, S, U, T, N, Rec	OS+RFS	MV	Nuc: OS: 2.74 RFS: 2.95 Cyt: OS: 0.80 RFS: 0.71	Nuc: OS: 1.29-5.79 RFS: 1.40-6.24 Cyt: OS: 0.34-1.88 RFS: 0.30-1.66
Cho S. [74]	SVV	FFPE	IHC	antibody 1:50	>25%	34.5	Cyt	G, HT	DFS	MV	2.21	1.01-5.65
Yang D.X. [87]	SVV	FFPE	IHC	Goat, Santa Cruz Biotechnology,1:100	Score ≥ 2 (staining intensity + percentage)	65	Cyt	A, S, HT, U, TS, G, N	DSS	UV (SC)	2.72	1.47-5.04
Chen Y.Q. [88]	SVV	FFPE	IHC	Goat mAb, Santa Cruz	NA	81.7	Cyt > Nuc	A, S, G, U, N	NA	NA	NA	NA

				Biotechnology, 1:200								
Ikehara M. [89]	SVV	FFPE	IHC	Goat pAb, Santa Cruz Biotechnology1: 200	>10%	51.9	NA	A, Vi, Li, N, HT, CEA	OS	UV (SC)	4.42	1.81-10.80
Fan C.F. [90]	SVV	FFPE	IHC	Rabbit pAb, RB-1629, Thermo Fisher, 1:100	Score ≥ 2 (staining intensity + percentage)	48.7	Cyt	A, S, HT, G, U, TS, N	OS	MV	2.31	1.35-4.02
Yu S. [91]	SVV	FFPE	IHC	Goat pAb, Cell Signaling, 1 :100	Score ≥ 4 (staining intensity + percentage)	68.8	Cyt	A, S, Smo, TS, G, U, N	NA	NA	NA	NA
Grossi F. [93]	SVV	FFPE	IHC	Rabbbit pAb, Abcam, ab 469, 1:50	>50%	71.3	Cyt	NA	OS	MV	1.61	0.94 -2.77
Wang X.Y. [94]	SVV	FFPE	FISH	NA	Score > 2 (intesnity + percentage of staining)	66.7		U, G, N, HT	NA	NA	NA	NA
Xia R. [95]	SVV	FFPE	IHC	Santa Cruz Biotechnology, 1:100	Median Score (staining intensity + percentage)	NA	Nuc	NA	OS	MV	2.06	1.06-4.01
Liang Y. [55]	Livin	FFPE	IHC	Rabbit, Abcam, 1:500	Score ≥ 4 (staining intensity + percentage)	57.8	Cyt	A, S, G, T, N, M, U	OS	MV	0.57 (neg. vs. pos.)	0.34-0.95
Ferreira C.G. [96]	XIAP	FFPE	IHC	Mouse mAb, Clone 2F1, MBL, 1:75	≥20%	43.8	Cyt	A, S, HT, G, T, N, U	OS	MV	0.63	0.42-0.95
Hofmann H.S. [97]	XIAP	NA	RT-PCR	NA	Median increase + 60%	50		HT, T, N, U	NA	NA	NA	NA

Dong X. [53]	BIRC6	FFPE	IHC	Rabbit pAb, Novus Biologicals, 1:100	Score ≥ 1 (staining intensity + percentage)	53.2	Cyt	(A, S, HT, T, N, U)[NE]	RFS	MV	2.23	1.08-4.60
Gharabaghi M.A. [98]	BIRC6	FFPE	IHC	Rabbit pAb, Abcam	NA	75	NA	A, S, T, N, G	OS	MV	3.42	1.84-12.40
Vischioni B. [75]	SVV	FFPE	IHC	Rabbit pAb, ab469, 1:2000	>5%	88.7	Nuc + Cyt	A, S, HT, G, U, TC, RC, M	OS+RFS	MV	OS: 0.45 RFS: 0.47	OS: 0.22- 0.90 RFS: 0.22-0.98
Wang M. [76]	SVV	FFPE	IHC	pAb, Neomarkers, 1:200	Score ≥ 2 (staining intensity + percentage)	53.3	Cyt	A, S, TS, Smo, HT, N, G	OS	MV	1.95	1.35-2.84
Wu Y.K. [77]	SVV	FFPE	IHC	mAb, 1:1000	H-score > 6 (staining intensity + percentage)	54.2	Cyt+Nuc	(A)[NE], S, T, N, M, U, HT, TC, ECOG, Smo, nCT	OS	MV	6.16	1.44-26.32
Yamashita S.I. [78]	SVV	FFT	RT-PCR	NA	Expression > 1	59.6		A, S, (Smo)[NE], T, N, HT	OS	UV	0.62 (neg. vs. pos.)	0.22-1.75

Akyürek N. [50]	SVV	FFPE	IHC	Rabbit pAb, Neomakers, 1 :100	10%	64.1	Cyt > Nuc	A, S, HT, T, N, M, U, Smo, p53	OS	UV (SC)	2.96	1.66-5.25
Cho H.J. [79]	SVV	FFPE	IHC	Rabbit mAb, clone 71G4B7, Cell Signaling, 1:800	Score ≥ 2 (staining intensity + percentage)	71.7[1] 88.7[2]	NA	NA	OS	MV[1] UV[2]	2.53 1.23	1.04-6.16 0.57-2.65
Falleni M. [80]	SVV	FFT	RT-PCR	NA	25n	53		NA	OS	UV (SC)	1.09	0.50-2.38
Hirano H. [81]	SVV	FFPE	IHC	Rabbit pAb Novus Biologicals, 1:500	LI ≥ 3	43.2	Cyt + Nuc	S, HT, (BI, TS, A)[NE]	NA	NA	NA	NA
Kren L. [54]	SVV	FFPE	IHC	Goat pAb, Santa Cruz Biotechnology 1:40	intense diffuse, intense regional, moderate diffuse,	52.9	Cyt	NA	OS	UV (SC)	3.12	1.96-4.97

					moderate regional							
Porebska I. [82]	SVV	FFPE	IHC	Mouse mAb, clone 12C4, DakoCytomation, 1:50	>20%	53.4	Cyt > Nuc	N, T, U, HT	OS	UV (SC)	1.14	0.58 -2.25
Nakashima N. [83]	SVV	FFPE	IHC	Mouse mAb, sc-17779, Santa Cruz Biotechnology, 1:50	H-score >1 (staining intensity + percentage)	52.5	Nuc + Cyt	Smo, T, N, U, G, HT	OS	UV (SC)	1.88	1.00-3.53
Atikcan S. [84]	SVV	FFPE	IHC	Mouse mAb, Clone 4F7, Neomarkers, 1:50	>25%	Nuc: 48.3 Cyt: 96.6	Nuc + Cyt	NA	OS	MV	3.73	1.53-9.05
Huang W. [85]	SVV	FFPE	IHC	Rabbit pAb, RAB0536, Fuzhou Maixin Biotechnology, 1:100	Score > 3 (staining intensity + percentage)	73.8	Cyt	A, S, Smo, HT, G, U, T, N, M	OS+PFS	UV (SC)	OS: 1.78 PFS: 2.25	OS: 1.03-3.08 PFS: 1.05-4.84
Kim G.Y. [86]	SVV	FFPE	IHC	Rabbit pAb, NB500-201, Novus Biologicals, 1:1000	Score ≥ 12 (staining intensity + percentage)	ADC Nuc: 66.7 Cyt: 72 SCC Nuc: 88.7 Cyt: 76.8	Nuc + Cyt	A, S, HT, G, T, N, M, U	OS	MV	ADC: Nuc: 0.51 Cyt: 4.51 SCC: Nuc: 0.96 Cyt: 2.05	ADC: Nuc: 0.09-2.89 Cyt: 1.71-11.93 SCC: Nuc: 0.31-2.96 Cyt: 1.15-3.58
Li C. [92]	SVV	FFPE	IHC	Rabbit mAb, ab134170, Abcam	H-score (staining intensity + percentage)[ND]	65.3	NA	NA	OS	UV	0.76 (neg. vs. pos.)	0.37-1.53
Sun L. [99]	SVV	FFPE	IHC	pAb, Santa Cruz Biotechnology	Score ≥ 2 (staining	57.6	NA	NA	OS	MV	0.86	0.55-1.28

	intensity + percentage)	

Abbreviations: S: sex; A: age; D: differentiation; N: lymph node metastasis; U: UICC stage; M: metastasis; HT: histological type; AT: adjuvant therapy; T: T stage; G: grading; Li: lymphatic invasion; Vi: venous invasion; Pn: perineural invasion; Smo: smoking; PS: performance status; ADC: adenocarcinoma; SCC: squamous cell carcinoma; Rec: recurrence; TT: tumor type; IASLC: international association for study of lung cancer; ECOG: eastern cooperative oncology group; Rt: resection type; Pi: pleura invasion; TS: tumor size; WL: weight loss; CR: clinical response; RC: response to chemotherapy; nCT: number of chemotherapy cycles; TC: type of chemotherapy; BI: Brinkman index; CEA: carcinoembryonic antigen; p53: p53 status; SNP: single nucleotide polymorphism; FFPE: formaline fixed paraffin embedded; FFT: fresh frozen tissue; LI: labeling index; Nuc: nuclear; Cyt: cytoplasmic; SVV: survivin; XIAP: X-linked inhibitor of apoptosis protein; BIRC6: baculoviral IAP repeat containing 6 (BRUCE); FISH: Fluorescence in situ hybridization; IHC: immunohistochemistry; RT-PCR: reverse transcription polymerase chain reaction; UV: univariate; MV: multivariate; DFS: disease free survival; DSS: disease specific survival; OS: overall survival; PFS: progression free survival; RFS: recurrence/relapse free survival; TFS: tumor free survival; SC: survival curve; NA: not available; ND: not defined; NE: not extractable; [1]: post-treatment; [2]: pre-treatment

Table S2. Association between XIAP/BIRC4 and clinicopathological variables

			Pooled Data (Random)			Test for Heterogeneity		
**Clinicopathological Variable**	**No. of Studies**	**Cases**	**OR**	**95% CI**	**P-value**	**Chi^2**	**P-value**	**I^2 (%)**
Sex (female/male)	1	144	1.35	0.56-3.26	NA	NA	NA	NA
UICC stage (I+II/III+IV)	2	178	0.50	0.17-1.45	0.20	0.74	0.39	0
T stage (T1+2/T3+4)	2	178	0.51	0.09-2.75	0.43	2.50	0.11	60
Differentiation (well+moderate/poor)	1	144	0.65	0.32-1.32	NA	NA	NA	NA
Lymph Node Metastasis	2	171	0.75	0.37-1.52	0.43	0.00	1.00	0

Table S3. Association between Livin/BIRC7 and clinicopathological variables

**Clinicopathological Variable**	**No. of Studies**	**Cases**	**OR**	**95% CI**
Sex (female/male)	1	90	1.27	0.55-2.96
Age	1	90	1.53	0.66-3.58
UICC stage (I+II/III+IV)	1	90	2.59	1.05-6.40
T stage (T1+2/T3+4)	1	90	1.52	0.57-4.07
Lymph Node Metastasis	1	90	2.66	1.13-6.30
Distant metastasis	1	90	1.48	0.13-16.94
Differentiation (well+moderate/poor)	1	90	1.24	0.28-5.54

Table S4. Association between BRUCE/BIRC6 and clinicopathological variables

**Clinicopathological Variable**	**No. of Studies**	**Cases**	**OR**	**95% CI**
Sex (female/male)	1	40	0.67	0.16-2.81
Age	1	40	3.50	0.75-16.28
T stage (T1+2/T3+4)	1	40	4.57	0.83-25.21
Differentiation (well+moderate/poor)	1	40	31.08	1.67-579.95
Lymph Node Metastasis	1	40	13.14	2.25-76.81

Table S5. HR and 95% CI according to the expression of IAP/BIRC family members in LUAD, LUSC and the combined histological subtypes (LUAD + LUSC) using TCGA datasets

IAP/BIRC family member	Overall survival (OS)			Disease free survival (DFS)		
	**LUAD**	**LUSC**	**Combined**	**LUAD**	**LUSC**	**Combined**
NAIP/BIRC1	0.61 (0.46 – 0.82)	1.4 (1.05 – 1.87)	0.92 (0.41 – 2.09); p = 0.85	0.68 (0.44 – 1.03)	1.36 (0.82 – 2.27)	0.91 (0.66 – 1.27); p = 0.59
cIAP1/BIRC2	1.93 (1.42 – 2.61)	0.88 (0.67 – 1.16)	1.30 (0.60 – 2.80); p = 0.51	1.62 (1.03 – 2.55)	1.71 (0.95 – 3.06)	1.65 (1.15 – 2.37); p = 0.006
cIAP2/BIRC3	1.71 (1.26 – 2.31)	1.41 (1.02 – 1.94)	1.56 (1.25 – 1.95); p < 0.0001	1.56 (0.99 – 2.46)	1.66 (0.97 – 2.84)	1.60 (1.13 – 2.27); p = 0.008
XIAP/BIRC4	0.86 (0.65 – 1.15)	0.85 (0.65 – 1.12)	0.85 (0.70 – 1.04); p = 0.11	1.53 (0.99 – 2.37)	1.72 (1.03 – 2.86)	1.61 (1.15 – 2.24); p = 0.005
Survivin/BIRC5	1.81 (1.33 – 2.47)	0.79 (0.59 –1.06)	1.19 (0.53 – 2.69); p = 0.67	1.51 (0.99 – 2.3)	1.71 (0.98 – 2.96)	1.58 (1.13 – 2.20); p = 0.007
BRUCE/BIRC6	0.83 (0.61 – 1.11)	0.78 (0.58 – 1.04)	0.80 (0.65 – 0.99); p = 0.04	1.55 (0.95 – 2.51)	1.55 (0.93 – 2.59)	1.55 (1.09 – 2.20); p = 0.01
Livin/BIRC7	0.68 (0.51 – 0.92)	1.21 (0.92 – 1.59)	0.91 (0.52 – 1.60); p = 0.74	1.31 (0.81 – 2.11)	0.68 (0.41 – 1.12)	0.96 (0.68 – 1.36); p = 0.82
Ts-IAP/BIRC8	0.83 (0.62 – 1.11)	0.72 (0.55 – 0.95)	0.77 (0.63 – 0.94); p = 0.009	0.63 (0.4 – 0.99)	0.74 (0.45 – 1.22)	0.68 (0.48 – 0.95); p = 0.02
